# Thermo-related degeneration of stumpy forms of *Trypanosoma brucei*, the pathogen of African sleeping sickness

**DOI:** 10.1007/s44307-025-00081-9

**Published:** 2025-09-23

**Authors:** Jia-Yi Luo, Ju-Feng Wang, Jiong Yang, Peng Zhang, Geoff Hide, De-Hua Lai, Zhao-Rong Lun

**Affiliations:** 1 MOE Key Laboratory of Gene Function and Regulation, State Key Laboratory of Biocontrol and Guangdong Provincial Key Laboratory of Aquatic Economic Animals, School of Life Sciences, Sun Yat-Sen University, Guangzhou, 510275 The People’s Republic of China; 2 Biomedical Research and Innovation Centre, School of Science, Engineering and Environment, University of Salford, Salford, M5 4WT UK; 3 Present Address. Southern Marine Science and Engineering Guangdong Laboratory (Guangzhou), Guangzhou, 511458 The People’s Republic of China

**Keywords:** *Trypanosoma brucei*, Stumpy form, Fever-related clearance, Mitochondrial dysfunction, Oxidative stress, Metabolic disruption

## Abstract

*Trypanosoma brucei*, the causative agent of African trypanosomiasis, develops from the long slender (LS) to the short stumpy (SS) form in the mammalian host. The SS trypanosomes are critical for transmission to the insect vector but face significant challenges within the vertebrate host. The role of the immune response in controlling the parasitaemia is well studied, however, the mechanism underpinning the rapid degeneration of SS trypanosomes during the first parasitaemic peak in mice remains somewhat elusive. We demonstrate that fever is a critical yet underexplored factor in facilitating the clearance of SS trypanosomes, suggesting that temperature may play a critical role in regulating the natural turnover of SS trypanosomes. The elevated body temperature correlates with the parasitaemic dynamics, accelerating SS trypanosome elimination in the mammalian host. The SS trypanosomes exhibited high thermo-sensitivity to elevated temperatures, accompanied with apoptosis-like events, mitochondrial damage and oxidative stress. Metabolomic profiling also revealed disruptions in glycolysis and the TCA cycle, shedding light on the processes in compromising the SS trypanosomes. Interestingly, antibodies during the acute phase did not directly cause SS trypanosomes death, but the combination of elevated temperature and antibodies enhanced the clearance of SS trypanosomes, highlighting the critical role of fever in eliminating the first parasitaemic peak. Our findings detail the mechanism of vulnerability of SS trypanosome to elevated temperatures and suggest that host fever serves as a neglected, but critical mechanism, for *T. brucei* SS trypanosome clearance.

**Supplementary Information:**

The online version contains supplementary material available at 10.1007/s44307-025-00081-9.

## Introduction

*Trypanosoma brucei (T. brucei)* is a parasitic protozoan responsible for African trypanosomiasis, commonly known as sleeping sickness in humans and nagana in animals (Buscher et al. 2017). *T. brucei* navigates between the mammalian host and the tsetse fly vector, undergoing morphological and metabolic transformations that are critical for its pathogenicity and survival (Vickerman 1985). Within the mammalian host, the bloodstream forms (BSFs) of this parasite exist primarily in two forms: the proliferative long slender (LS) and the non-dividing short stumpy (SS) form. The LS trypanosome is responsible for the rapid rise in parasitaemia, after which it differentiates into the SS trypanosome using a quorum-sensing mechanism (Mony and Matthews 2015; Reuner et al. 1997; Vassella et al. 1997). The differentiation to SS trypanosomes is a critical adaptation, prolonging the host survival time and enhancing transmission to the tsetse fly vector (Rico et al. 2013; Smith et al. 2017).

For *T. brucei* infection, one of the key challenges in addressing the pathogenicity is the clearance of trypanosomes within the mammalian host. It crucially depends on two distinct processes: host-mediated immune responses and intrinsic parasite turnover through natural degeneration. For clearance of parasites in the mammalian host, previous research has primarily addressed two mechanisms: the activity of trypanosome lytic factors (TLFs) (Raper et al. 1999; Rifkin 1978) and the role of antibody-related elimination (Black et al. 2010; Mugnier et al. 2015). Use of murine infection models demonstrated a significant parasitaemic peak in early infection (Magez et al. 2008; Rijo-Ferreira et al. 2018), followed by a chronic phase characterized by several lower fluctuations. The first parasitaemic peak is particularly severe, with a high parasite density that, if not effectively controlled through differentiation, can lead to host mortality (Lun et al. 2015; Sendashonga and Black 1986). While the murine model doesn’t have the TLFs, the elimination of the first high parasitaemia is now widely considered to be a result of IgM-triggered clearance (Black et al. 1983, 1986; Sendashonga and Black 1982). As far as we know, most of the trypanosomes are in the SS form during the remission of the first parasitaemic peak (Larcombe et al. 2023). As the transitional stage from mammalian host to insect vector, the SS trypanosomes exhibit sensitivity to residential temperature (Engstler and Boshart 2004) and can survive for around 4 days in the bloodstream (Savill and Seed 2004; Turner et al. 1995). Interestingly, the degeneration of SS trypanosomes during the first parasitaemic peak seems to be much faster than the natural turnover found in previous studies (Black et al. 1983). Clearly, some unexplained factors may contribute to the natural turnover of SS trypanosomes, within the mammalian host, especially during the first peak.

We also notice that infections with *T. brucei*, like many other pathogens, elicit febrile responses in patients (Kennedy 2013). However, the effect of fever on the natural turnover of SS trypanosomes has received limited attention. A few experiments mimicking fever have been carried out on LS trypanosomes, using heat shock, which revealed a thermosuppressive effect related to the fever (Ooi et al. 2020). In addition, there was a study which showed that temperature uplift was able to kill the insect procyclic forms (PCFs) (Droll et al. 2013). To the best of our knowledge, no studies have been systematically conducted on the impact of host fever on the dynamic degeneration of SS trypanosomes.

Here, we demonstrate that the host fever plays a critical role in compromising the viability of SS trypanosomes, thereby accelerating the clearance of the first parasitaemic peak in mice. We specifically assess the influence of antibodies and the elevated temperatures on the clearance of SS trypanosomes, explore the relationship with oxidative stress, apoptosis-like events, mitochondrial integrity and metabolomic disruptions, whilst emphasizing their association with cell viability.

## Results

### Correlation between parasitaemia and body temperature in early T. brucei infection

**Fig. 1:**
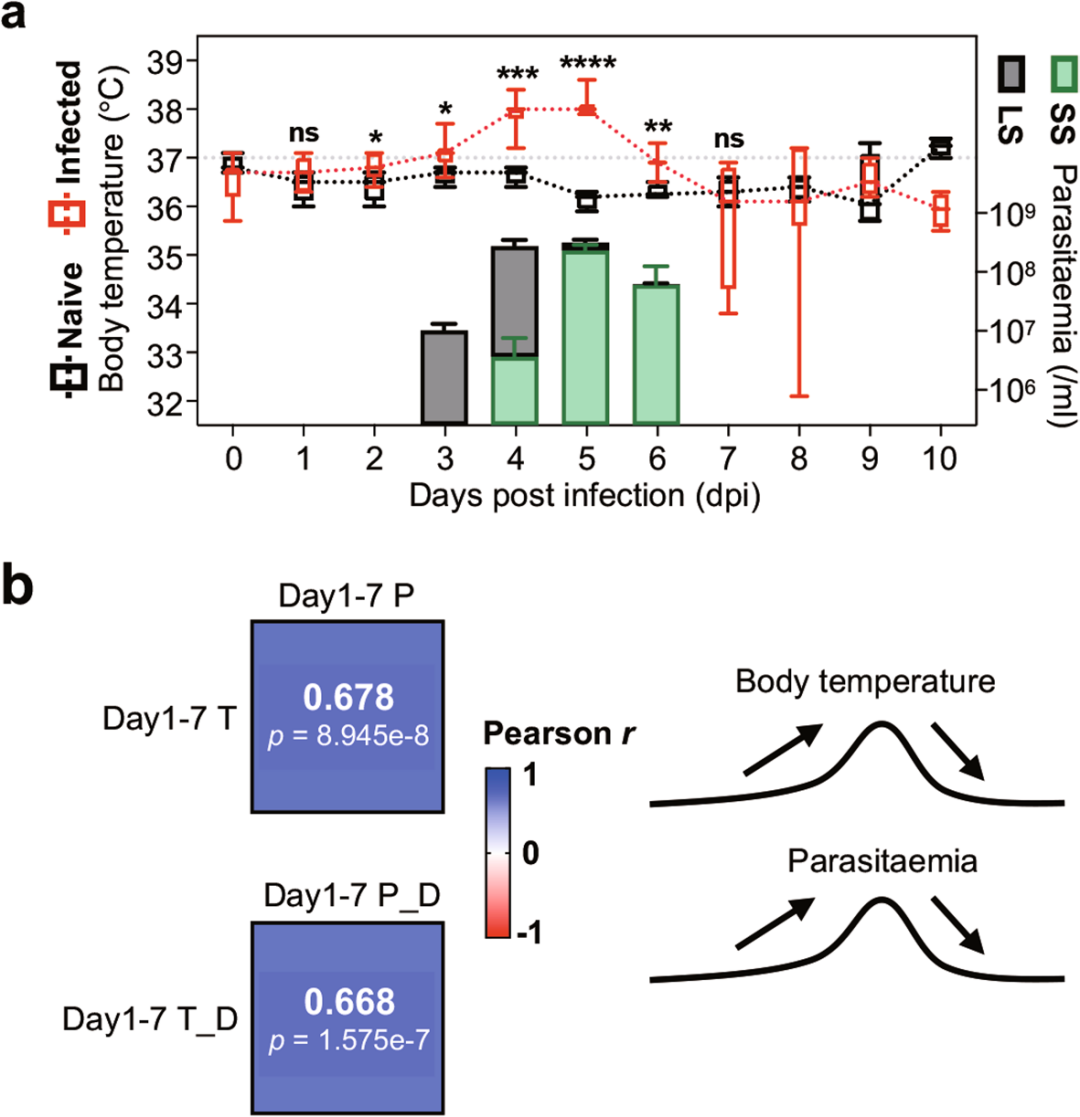
Parasitaemia and body temperature dynamics in the early stages of *T. brucei* infection. **a** Dynamics of the parasitaemia and body temperature in infected mice. The differentiation of trypanosomes was quantified using blood smears and microscopy. Results are presented as the median and interquartile range (IQR) for body temperature, and as the mean ± SD (for parasitaemia). The IQR is represented as a box spanning from the 25th to 75th percentiles, with whiskers extending to the minimum and the maximum values. Significance tests of body temperature were performed using a two-tailed unpaired Student’s *t*-test on the first 6 days (* *p* < 0.05, ** *p* < 0.01, *** *p* < 0.001, **** *p* < 0.0001), and using a two-tailed unpaired Welch’s *t*-test at 7 dpi (ns, *p* ≥ 0.05). **b** Pearson correlation analysis between parasitaemia and body temperature (P for parasitaemia, T for body temperature) and their daily differences (T_D & P_D) to the last day

Previous studies, for example in a rabbit model, have noticed and suggested a possible correlation between the parasitaemia and the body temperature (Toth et al. 1994). We set out to better understand the dynamic interaction between host temperature and parasitaemia, especially on the composition of different trypanosome populations, by conducting an experimental infection in C57BL/6J mice with pleomorphic *T. brucei* (Fig. S1a). The mortality events were systematically monitored daily throughout the study (Fig. S1b). In the infected group, the parasitaemia was firstly detected approximately 3 days post-infection (dpi), with the long slender (LS) trypanosomes differentiating into the short stumpy (SS) trypanosomes by 4 dpi, coinciding with the onset of fever (Fig. 1a). Both the parasitaemia and the body temperature of infected mice peaked on 4 and 5 dpi, with the parasite population predominantly consisting of SS trypanosomes (nearly 74%) on 5 dpi. Additionally, the body temperature of the infected group showed the most significant difference compared to the naive group on these two days. The parasitaemia started to decline on 6 dpi, with the trypanosome population represented by nearly 97% of the SS forms, and sharply dropped to the undetectable levels by 7 dpi. In this same time frame, the body temperature of infected mice gradually returned to the baseline levels. These observations suggested a potential linkage between the fluctuations in host body temperature and the dynamics of parasitaemia.

To further quantify this relationship, we performed a Pearson correlation analysis on the collected data (Fig. 1b). The analysis revealed a strong positive correlation between the parasitaemia and body temperature during the first 7 days of infection (1–7 dpi), with a correlation coefficient (*r*) of 0.678 (*p* = 8.945e-8), indicating a synchronous fluctuation in both parameters. In addition, the correlation coefficient (*r*) between the daily difference of these two groups was 0.668 (*p* = 1.575e-7). Since it is acknowledged that host fever serves as a key mechanism in the elimination of pathogens, here, we tried to further explore the relationship between the host body temperatures and the parasitaemic dynamics (Table S1). Multiple linear regression analysis was performed to investigate the associations between the dependent variable (Day567_P) and five time-zone predictors (Day123_T, Day234_T, Day345_T, Day456_T, Day567_T). Multicollinearity diagnostics indicated acceptable levels of variance inflation factors (VIFs) across predictors (range 1.397 to 2.917), suggesting no severe multicollinearity. Additionally, residual analyses confirmed normality assumptions (Shapiro–Wilk *W* = 0.9878, *p* = 0.9926; Anderson–Darling *A*^2*^ = 0.1624, *p* = 0.9349), supporting the validity of the regression model. The overall model was statistically significant (F = 6.242, *p* = 0.0025), explaining 67.54% of the variance in the dependent variable (R^2^ = 0.6754). Among the predictors, only Day345_T demonstrated a significant negative association with Day567_P (β < 0, *p* = 0.0457), while other predictors showed no statistically significant effects. Notably, when the dependent variable was specified as Day567_SS, the *p* value for Day345_T increased slightly to 0.0548, also approaching the conventional threshold of statistical significance. These results highlight Day345_T as a critical time-zone predictor influencing the Day567_P level (also Day567_SS), while the other variables did not contribute significantly to the model.

Collectively, these findings demonstrate a robust parallel correlation between the parasitaemia and body temperature during the initial stages of *T. brucei* infection (up to 7 dpi) and especially supporting the relationship between host fever and the degeneration of SS trypanosomes.

### Fever is the key mechanism in accelerating the clearance of short stumpy trypanosomes

Given the strong positive correlation between the parasitaemia and the fluctuations of body temperature, we postulated that host fever might play a crucial role in the SS trypanosome elimination. The SS trypanosomes were purified from infected mice on 6 dpi (Fig. S2). As described in previous studies (Czichos et al. 1986; Engstler and Boshart 2004), the thermal sensitivity of SS trypanosomes was also confirmed in our study in HMI-9 medium (Fig. S3a, b). Briefly, the SS trypanosomes were cultured at 27 °C in HMI-9 medium, maintained and experienced differentiation into the procyclic forms (PCFs) as confirmed by morphology. While cultured at 37 °C in HMI-9 medium, the SS trypanosomes naturally turned over for nearly 4 days. Also, as further confirmation of the natural degeneration of SS trypanosomes at bloodstream temperatures was evidenced by the PAD1:P2A:tdTomato cell line to track the expression of PAD1 protein (Fig. S3c, d, e; Fig. S4).

**Fig. 2:**
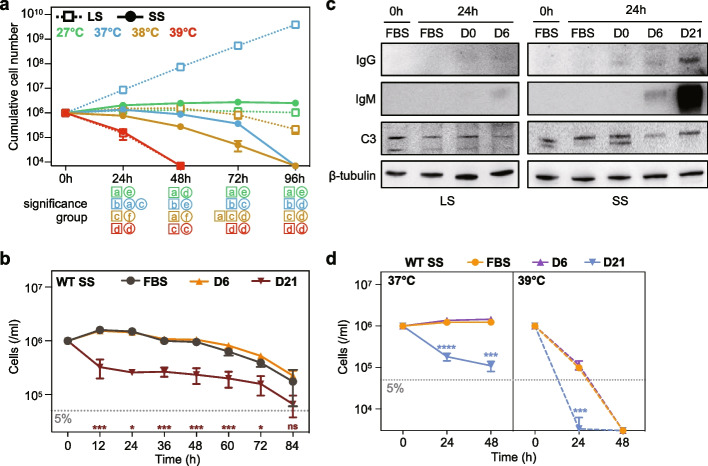
Effect of antibodies and temperature on the clearance of long slender and short stumpy trypanosomes. **a** Comparison of death of the LS and SS trypanosomes by treatment with different temperatures in HMI-9 medium. The initial cell concentration was 1 × 10^5^/ml for LS trypanosomes and 1 × 10^6^/ml for SS trypanosomes. Data were presented as cumulative cell number. Statistical analysis was performed by two-way ANOVA with post-hoc multiple comparisons. Raw *p*-values from multiple comparisons were adjusted using the original false discovery rate (FDR) method of Benjamini and Hochberg. Groups that do not share the same lowercase letter below each time point are significantly different after FDR correction (adjusted *p* < 0.05). **b** The suppression effect of antibodies on the SS trypanosomes at 37 °C in HMI-9 medium. Plasma from the uninfected (D0), the day-6 infected (D6) and the day-21 infected mice (D21) were added to HMI-9 medium. **c** The detection of parasite-bounded IgG, IgM and complement C3 on the LS and SS trypanosomes following co-culturing with D0 plasma, D6 plasma and D21 plasma for 24 h at 37 °C in HMI-9 medium. **d** Combination treatment of antibodies and high temperature applied to the SS trypanosomes in HMI-9 medium. Results are shown as a mean ± SD. For panel **b** and **d**, significance tests comparing the cell number of SS trypanosomes of D21 group with the FBS control group at the same day were performed using a two-tailed unpaired Student’s *t*-test or a two-tailed unpaired Welch’s *t*-test (ns *p* ≥ 0.05, * *p* < 0.05, ** *p* < 0.01, *** *p* < 0.001, **** *p* < 0.0001)

Interestingly, the natural turnover of SS trypanosomes seemed to be faster in vivo (2 or 3 days) than that in vitro (3 or 4 days). Given that the mice exhibited fever symptoms during early infection, we wanted to explore the sensitivity of SS trypanosomes to the elevated temperatures such as 39 °C, a realistic temperature recorded in a previous study (Rijo-Ferreira et al. 2018). As shown in Fig. 2a, the mortality rate of SS trypanosomes progressively increased with the rise of culture temperatures from 37 °C to 39 °C in HMI-9 medium. This suggests that fever could be an effective and essential mechanism for accelerating the elimination of SS trypanosomes within the host. Although both the LS and SS trypanosomes exhibited similar rates of degeneration when exposed to long-term (over 72 h) heat treatment at 38 °C, only the SS trypanosomes declined during the first 48 h. This suggests that the SS trypanosomes are, to some extent, more thermosensitive than the LS trypanosomes. These results suggest that host fever is more of a burden on the naturally degenerating SS trypanosomes than on the proliferating LS trypanosomes. At the same time, the effect of antibodies on trypanosomes during the rapid elimination of the first parasitaemic peak should be evaluated carefully. Co-culturing the SS trypanosomes with efficient antibodies (the plasma from infected mice at 21 dpi, D21 plasma) in HMI-9 medium led to a rapid decrease in their numbers, indicating that antibodies have a potent inhibitory effect (Fig. 2b; Fig. S5a). Interestingly, however, antibodies alone were insufficient for the complete elimination of SS trypanosomes, compared to the 100% elimination for LS trypanosomes (Fig. S5b), as previously described (McLintock et al. 1993). In the meantime, the plasma from infected mice at 6 dpi (D6 plasma) had no killing effect on the LS and SS trypanosomes with the presence of a little IgM antibody and complement C3 (Fig. 2b; Fig. S5b). To further validate these results, we collected the LS and SS trypanosomes following 24-h co-culture with plasma from infected mice and successfully detected parasite-bound IgG (D21 & SS), IgM (D6 & LS, D6 & SS) and complement C3 (Fig. 2c). Also, a combined treatment of elevated temperatures and D21 plasma could eradicate > 99.5% SS trypanosomes within 24 h in HMI-9 medium, while either approach alone left 15% ~ 18% of the SS trypanosomes alive (Fig. 2d). This indicates that the well-described clearance activity of IgM and C3 may not be the only factor in the elimination of the first parasitaemic peak. Instead, it may also be due to the natural degeneration of the SS trypanosomes themselves and their sensitivity to the elevated temperatures resulting from host fever.

### Impact of the elevated temperature on the differentiation of short stumpy trypanosomes to procyclic forms

**Fig. 3:**
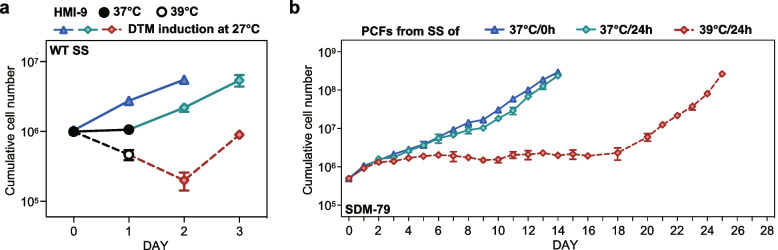
Alterations in the infectivity of short stumpy trypanosomes to the procyclic forms in response to elevated temperature. **a** Cumulative cell numbers of the SS trypanosomes cultured under different temperature conditions in HMI-9 medium and subsequently induced to differentiate into the PCFs in DTM medium at 27 °C for 48 h. **b** Proliferation of the PCFs derived from the SS trypanosomes initially cultured at 0 h (blue triangles), 37 °C for 24 h (green diamonds), and 39 °C for 24 h (red diamonds) in SDM-79 medium at 27°C. Results are shown as a mean ± SD

To evaluate if the SS trypanosomes retained their ability to differentiate into the PCFs after heat treatment, we compared the freshly isolated SS trypanosomes (37°C/0h) with those treated at 37 °C for 24 h in HMI-9 medium. Both the treated and untreated SS trypanosomes exhibited immediate and robust responses to DTM medium induction (which promotes the differentiation to PCFs) at 27 °C, as indicated by a significant increase in cell density for two days (Fig. 3a). In the case of the SS trypanosomes subjected to 39 °C for 24 h in HMI-9 medium, they showed a substantial reduction in cell numbers during the treatment. Following the DTM induction at 27 °C, these cells continued to decrease in their cell density on the first day and began growing on the second day. Subsequently, we transferred the three cell populations to SDM-79 medium at 27 °C and found the former two displayed similar proliferative capacities, initiating immediate and stable growth, while the cells from 39 °C group experienced a prolonged lag phase (Fig. 3b), until a notable growth from day 20, suggesting a severe impairment by exposure to fever heat.

These findings suggest that while the SS trypanosomes at 37 °C largely preserve their ability to differentiate into the PCFs, exposure to 39 °C compromises this capacity, leading to delayed recovery and impaired growth.

### High temperature triggers apoptosis-like events, mitochondrial damage and vesicular alterations in short stumpy trypanosomes

**Fig. 4:**
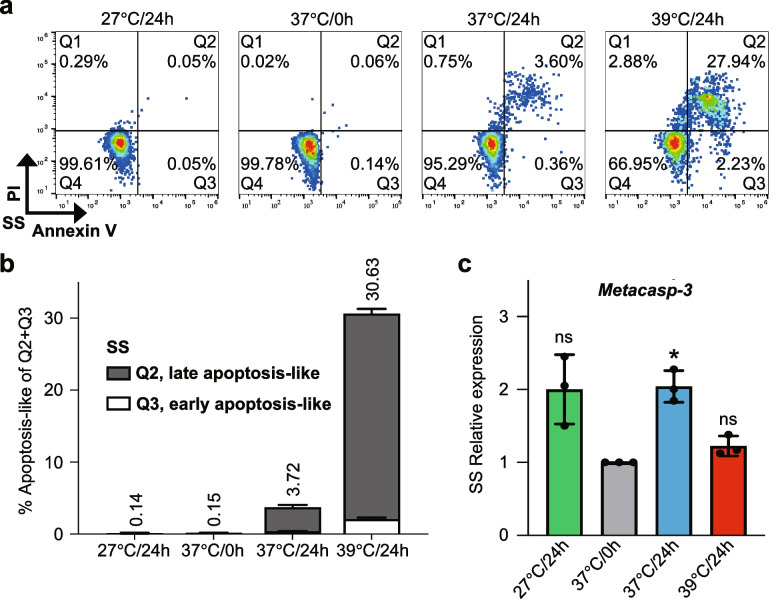
Evaluation of apoptosis-like events in short stumpy trypanosomes at the designated temperatures. **a** Analysis of phosphatidylserine exposure in the SS trypanosomes after 24-h culture by flow cytometry at different temperatures in HMI-9 medium. **b** Different percentages of apoptosis-like processes in the SS trypanosomes. **c** Relative expression levels of the apoptosis-like associated gene *Metacasp-3* in the SS trypanosomes at different temperatures in HMI-9 medium using RT-qPCR. The data are normalized to the reference gene *28S rRNA*. Results are shown as a mean ± SD. The experiments were conducted in three independent biological replicates. Statistical significance was assessed using a two-tailed paired Student’s *t*-test, with significant differences indicated by asterisks (*p < 0.05)

The elevated temperatures have been implicated in inducing cellular stress responses including apoptosis-like events. Also, trypanosomes now are considered to exhibit apoptosis-like processes (Menna-Barreto 2019). In the SS trypanosomes, apoptosis-like events were observed under heat stress conditions in HMI-9 medium (Fig. 4a, b). While an apparent increase in apoptosis-like events was observed in SS trypanosomes after 24-h incubation at 37 °C (3.72%) and at 39 °C (30.63%), almost no apoptosis-like events were observed in the SS trypanosomes cultured at 27 °C (0.14%). By comparison, LS trypanosomes presented no apoptosis-like events at 27 °C, 37 °C and 39 °C (Fig. S6a).

To determine which pathway is mainly involved in the heat-induced apoptosis-like events, we analyzed the expression levels of genes associated with the apoptosis-like processes (Fig. 4c; Fig. S6b). We observed a significant upregulation (~ twofold change) of a mitochondrial-associated apoptosis-like gene, *Metacasp-3* (Smirlis et al. 2010), at 37 °C after 24 h of incubation (Fig. 4c). By contrast, another apoptosis-like associated gene, *tSNAP42* via spliced-leader RNA silencing (Goldshmidt et al. 2010), showed negligible upregulation (Fig. S6b). In the meantime, SS trypanosomes also exhibited a trend of *Metacasp-3* gene up-regulation at 27 °C, approaching significance (*p* = 0.0678) under a two-tailed paired Student’s *t*-test. We propose that this phenomenon may result from the temperature-induced differentiation of SS trypanosomes into the PCFs at 27 °C as evidenced by the elevated apoptosis-like events observed during DTM induction (Fig. S6c). These data suggest that the apoptosis-like events observed in the SS trypanosomes are mainly driven by mitochondria.

**Fig. 5:**
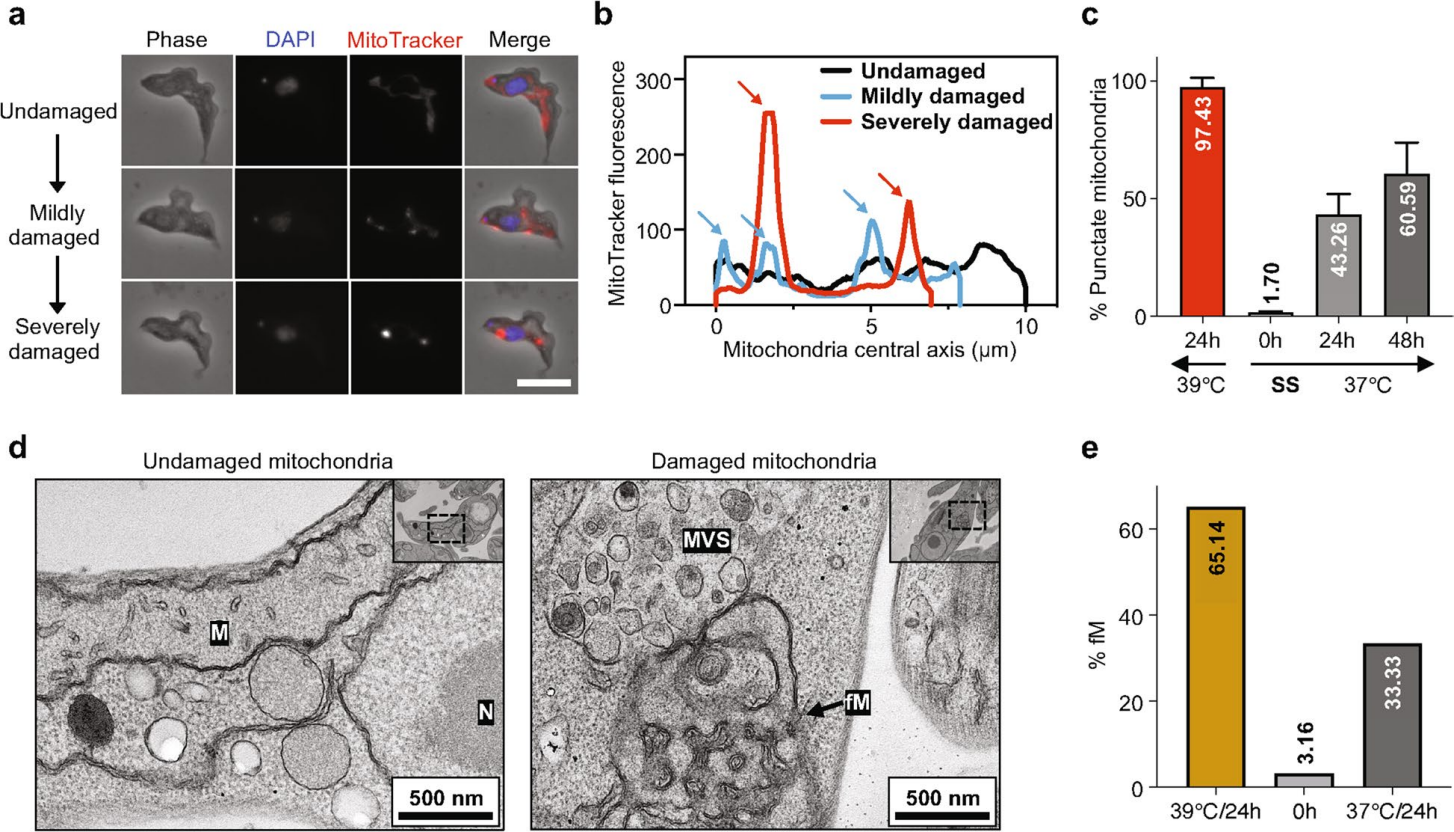
Mitochondrial alterations in short stumpy trypanosomes at different temperatures.** a** Fluorescence imaging of the SS trypanosome mitochondria during high temperature culture in HMI-9 medium, showing punctate aggregation of mitochondria of three types. Bar, 5 µm. **b** Fluorescence intensity profiles of central axis mitochondria in the SS trypanosomes post heat treatment using ImageJ. The arrows indicate the punctate mitochondrial aggregates in panel **a** visualized by MitoTracker staining. **c** The proportion of damaged mitochondria at various temperatures and treatment durations in HMI-9 medium. **d** Ultrastructural changes in mitochondria and vesicles of the SS trypanosomes post heat treatment using TEM. M, mitochondrion; fM, fenestrated mitochondrion; MVS, multivesicular structures; N, nucleus. **e** Percentage of fM of the SS trypanosomes following heat treatment in HMI-9 medium. Results are shown as a mean ± SD

Considering the apoptosis-like events and the significant up-regulation of *Metacasp-3* in the SS trypanosomes post heat treatment, we assessed whether mitochondria from SS trypanosomes were damaged. Three types of mitochondria from SS trypanosomes were observed: undamaged, mildly damaged and severely damaged (Fig. 5a). Unlike the smooth undamaged mitochondria, the damaged mitochondria exhibited aggregation of MitoTracker dye, forming more punctate structures. The quantitative fluorescence intensity profiles along the central axis of the mitochondria also confirmed these aggregations (indicated by arrows in Fig. 5b). At 37 °C, the proportion of damaged mitochondria rose gradually over the course of incubation, reaching approximately 43% by 24 h and about 60% by 48 h (Fig. 5c). While at 39 °C, the punctate aggregation was clearly observed in over 95% of the SS trypanosomes by 24 h of incubation. These results directly confirmed that exposure to high temperatures accelerates mitochondrial damage in the SS trypanosomes.

In addition, obvious damage of the SS trypanosomes was also confirmed by transmission electron microscopy (TEM), particularly the fenestrated mitochondrial structures (Fig. 5d). Additionally, the multivesicular structures were also found in the post-heat treated SS trypanosomes. This response may also indicate a potential stress-induced degradative pathway, such as autophagy, supported by the slight increase in *TbAtg8.2* expression (Fig. S6b). The percentage of fenestrated mitochondria became evident by 24 h of incubation at 37 °C and accumulated more rapidly at 39 °C (Fig. 5e).

These results demonstrate that the elevated temperatures accelerate apoptosis-like events in the SS trypanosomes, with notable mitochondrial damage.

### High temperature triggers oxidative stress and mitochondrial membrane permeabilization in short stumpy trypanosomes

**Fig. 6:**
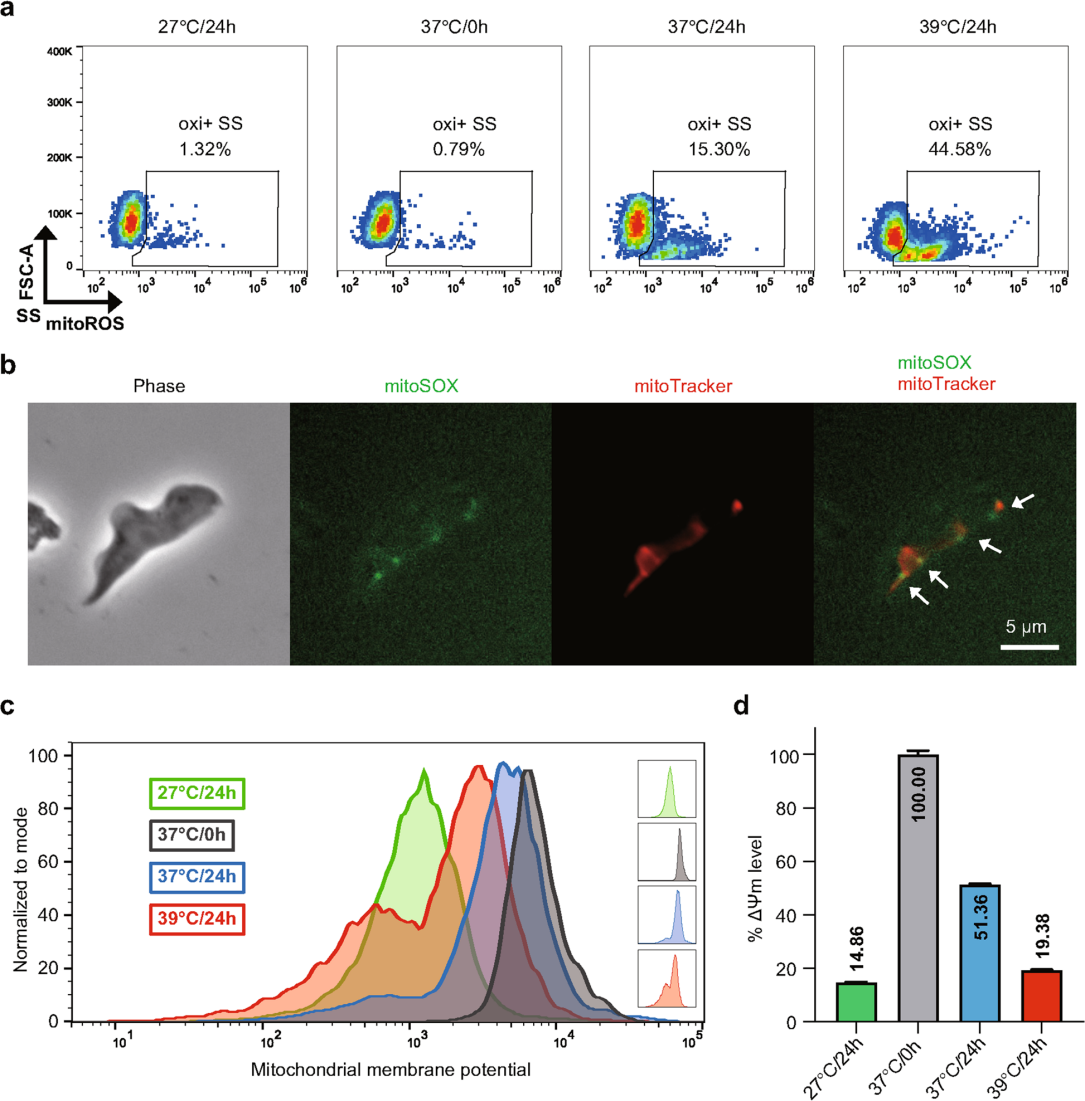
Evaluation of oxidative stress and mitochondrial membrane potential in short stumpy trypanosomes.** a** Flow cytometry fluorescence histograms of mitochondria ROS levels in the SS trypanosomes after 24-h incubation at different temperatures in HMI-9 medium. The gating of oxi + SS refers to those trypanosomes with oxidative stress. **b** Fluorescence images of MitoSOX (green, refers to mitoROS) and MitoTracker (red, refers to mitochondria) in the SS trypanosomes post heat treatment (39°C) with co-localization indicated by white arrows. **c** Histograms showing mitochondrial membrane potential in the SS trypanosomes after 24-h incubation at different temperatures in HMI-9 medium. **d** Quantification of mean fluorescence intensities for mitochondrial membrane potential in the SS trypanosomes after 24-h incubation at different temperatures in HMI-9 medium. Results are shown as a mean ± SD

Given these mitochondrial alterations, we believe that the exposure of SS trypanosomes to the elevated temperatures might lead to cellular stress. Our results indeed demonstrated that the SS trypanosomes accumulated mitochondrial ROS (mitoROS), when exposed to high temperatures (Fig. 6a). At 37 °C, the trypanosomes with oxidative stress (oxi + SS) rose from 0.79% to 15.30% within 24 h of incubation. This suggests that the SS trypanosomes experience oxidative stress at bloodstream temperatures. Furthermore, the SS trypanosomes at 39 °C exhibited a greater mitoROS accumulation, with 44.58% oxi + SS trypanosomes. In contrast, however, the SS trypanosomes at 27 °C showed little mitoROS accumulation, with only 1.32% oxi + SS trypanosomes. Furthermore, the intracellular localization of accumulated ROS coincided with the mitochondrial damage (Fig. 6b), indicating a strong correlation between the ROS stress and mitochondrial damage. On the other hand, the LS trypanosomes presented no oxidative stress within mitochondria when facing heat treatment (Fig. S7a).

To further assess mitochondrial function, we thus measured the mitochondrial membrane potential (ΔΨm) of SS trypanosomes. The freshly isolated SS trypanosomes exhibited a decreasing ΔΨm compared to the same freshly isolated LS trypanosomes (Fig. S7b). The SS trypanosomes cultured at 27 °C, 37 °C, and 39 °C for 24 h all exhibited mitochondrial membrane permeabilization, with a decline in ΔΨm, significantly reducing to 14.86%, 51.36% and 19.38%, respectively (Fig. 6c, d). Clearly, the SS trypanosomes incubated at 39 °C lost their mitochondrial membrane potential more rapidly compared to that of at 37 °C, as well as accumulated more oxidative stress.

In brief, exposure to the elevated temperatures accelerates oxidative stress and mitochondrial membrane permeabilization in the SS trypanosomes and are closely associated with heat-induced structural damage.

### Fever-induced metabolic disruptions in short stumpy trypanosomes

**Fig. 7:**
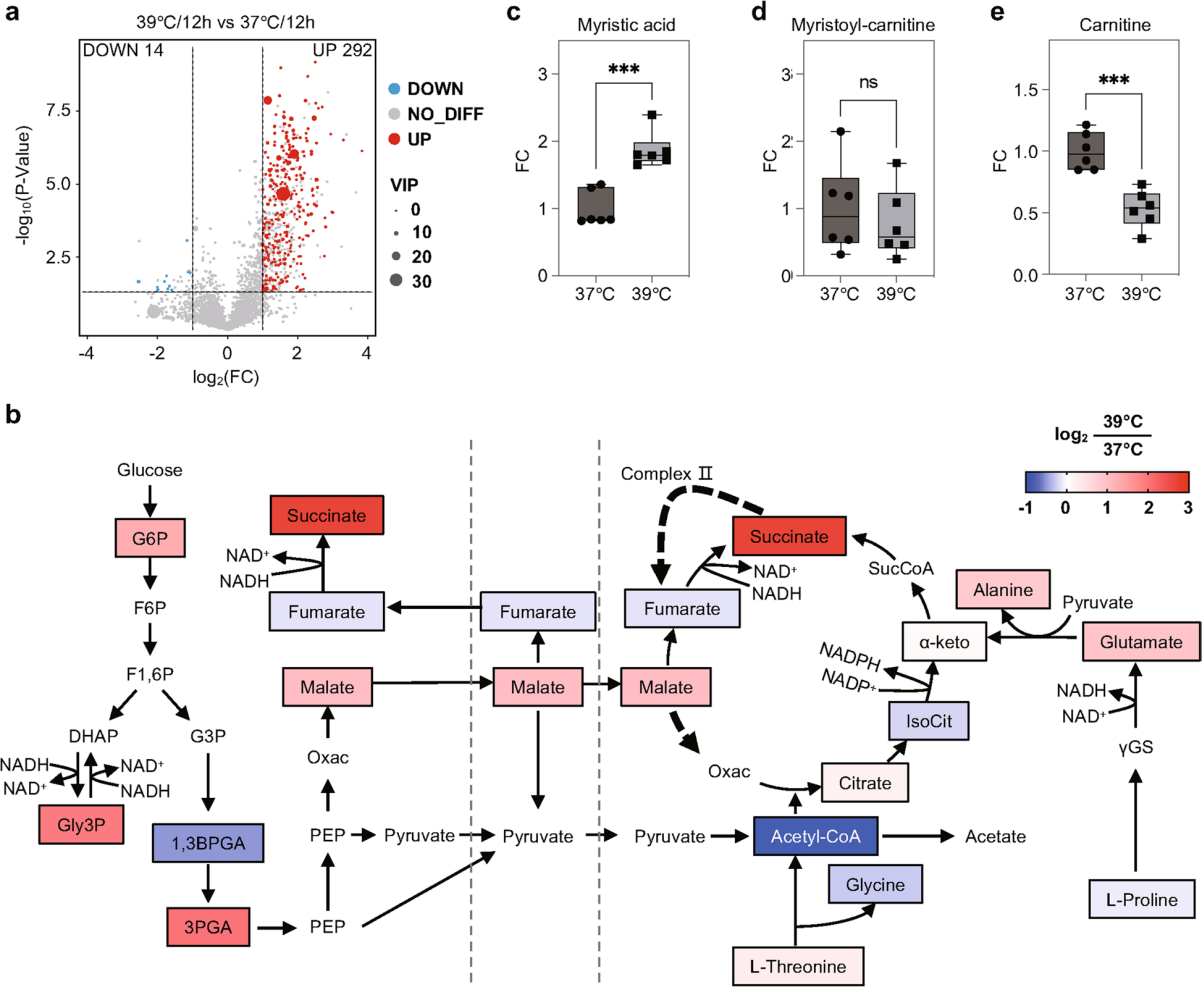
Metabolic disruptions in short stumpy trypanosomes incubated at different temperatures. **a** Volcano plot displaying differential metabolites in the ex vivo SS trypanosomes incubated in vitro at 39 °C compared to 37 °C for 12 h in HMI-9 medium. Metabolites with a fold change > 2 or < 0.5, a *p*-value from the *t*-test < 0.05 and a VIP score ≥ 1 were considered significantly different between the two groups. **b** Changes in glycolysis and the TCA cycle observed in the SS trypanosomes. The color represents the log2 fold change in metabolites between these two temperatures. G6P, glucose-6-phosphate. F6P, fructose-6-phosphate. F1,6P, fructose-1,6-biphosphate. DHAP, dihydroxyacetone phosphate. Gly3P, glycerol-3-phosphate. G3P, glyceraldehyde-3-phosphate. 1,3BPGA, 1,3-bisphosphoglycerate. 3PGA, 3-phosphoglycerate. PEP, phosphoenolpyruvate. Oxac, oxaloacetate. IsoCit, isocitrate. α-keto, α-ketoglutarate. γGS, γ-glutamate semialdehyde. Levels of myristic acid (**c**), myristoyl-carnitine (**d**) and carnitine (**e**) in the SS trypanosomes at 37 °C and 39°C. Results are shown as median ± IQR (Inter-quartile range). Statistical significance was assessed using a two-tailed unpaired Student's *t*-test (ns *p* ≥ 0.05, *** *p* < 0.001)

Understanding the metabolic disruptions in the SS trypanosomes during fever-induced stress is crucial for elucidating the molecular mechanisms underlying their accelerated degeneration. Given the well-established co-occurrence of glycolysis and a partially activated TCA cycle in the SS trypanosomes, we collected ex vivo SS trypanosomes after in vitro incubation at 37 °C and 39 °C, and analyzed them using untargeted metabolomics. Due to technical issues, we were unable to collect enough samples for the 24-h experiment. Therefore, we performed the metabolomic analysis on the 12-h samples. A total of 22,606 metabolites were detected, with the majority of differences between these temperature conditions observed under the negative ionization mode (Fig. S8a, b, c). Among these, 306 metabolites exhibited significant differential abundance (Fig. 7a; Fig. S8d).

Focusing on glycolysis and the TCA cycle, succinate was demonstrated to have the most notable accumulation, with its level being approximately six times higher at 39 °C than that at 37 °C (Fig. 7b; Fig. S8e, f; Table S2). Along the glycolysis and TCA cycle pathway to succinate, the intermediate metabolite fumarate was observed to be slightly lower in the SS trypanosomes incubated at 39 °C, corroborating the observed accumulation of succinate in the SS trypanosomes under febrile conditions. In other pathways to succinate, minimal changes were detected in citrate levels between these two temperatures, as well as α-ketoglutarate. The coupled accumulation of alanine and glutamate at 39 °C, suggests a significant inhibition of catalytic flux in these directions at elevated temperatures like 39°C. Furthermore, the substantial accumulation of glucose-6-phosphate (G6P) and 3-phosphoglycerate (3PGA) also suggests a disruption in both energy yield and utilization under 39 °C conditions.

We also detected the lipids and lipid-like molecules and found that an accumulation of myristic acid (C14:0) was detected at 39 °C (Fig. 7c). As the key component of the bloodstream trypanosomal glycosylphosphatidylinositol (GPI), the myristic acid levels were approaching two fold at 39 °C compared to that at 37 °C, while the carnitine-conjugated form remained unchanged. We believe that this resulted from the remodeling of myristoyl-GPI into GPI with a longer fatty acid moiety to prepare for the PCF stage, e.g. C16/18-GPI. Though the release of myristic acid was accelerated at 39 °C, its degradation by fatty acid β-oxidation was not likely (Fig. 7d), as evidenced by a significant downregulation of carnitine at 39 °C (Fig. 7e).

In general, these findings reveal substantial metabolic disruptions in the SS trypanosomes when they were exposed to host fever, suggesting that the elevated temperatures impair key pathways involved in energy production in these parasites.

## Discussion

Fever is a common clinical symptom observed in both patients and animals during early *T. brucei* infections (Liu et al. 2018; Wellde et al. 1989). In this study, a dramatic uplift of body temperature was observed just prior to the remission of the first parasitaemic peak, suggesting a strong association between the fever and trypanosome clearance. Notably, this study is the first to decisively illustrate the mechanisms underlying the nature of turnover of short stumpy (SS) trypanosomes. We propose that the host fever is the critical factor for accelerating the rapid remission of the first parasitaemic peak, which is dominated by SS trypanosomes.

Our results showed that the SS trypanosomes rapidly died at temperatures as high as 39 °C but also exhibited slower death rates at 37 °C as a function of their natural turnover in relation to temperature. To better understand this phenomenon, a comparison of the growth profiles of SS and LS trypanosomes was conducted at 37 °C as the optimal temperature for the rapid proliferation of LS trypanosomes. Generally, the elevated temperatures can induce oxidative stress within cells, manifested by the increased production of reactive oxygen species (ROS), which may disrupt the intracellular homeostasis, leading to cell death (Kirkinezos and Moraes 2001; Wargnies et al. 2021). In our experiments, boosting of ROS production was observed in the SS trypanosomes at 37 °C or above but not at their destination temperature of 27°C. This observation was consistent with our findings of apoptosis-like events. The boosting of ROS and apoptosis-like events suggests that the SS trypanosomes have not optimally evolved for life at 37 °C but are rather better adapted to 27 °C where their pre-adaptations for the insect gut environment are well-described. This is likely due to a partially activated TCA cycle and a non-canonical electron transport chain in the SS trypanosomes (Zikova 2022), which may generate ROS in a manner similar to the procyclic forms (PCFs). It is well established that the ROS levels are naturally high in the PCFs, as they rely on a fully functional electron transport respiratory chain for energy production in the nutrient limited vector (Michels et al. 2021). However, the SS trypanosomes, with a partially active TCA cycle, reside at a much higher temperature than the PCFs, making them vulnerable to the ROS-induced degeneration at 37°C. On the other hand, the SS trypanosomes may also retain a partially active anti-oxidant system similar to that of the LS trypanosomes. Indeed, lower expression levels of iron superoxide dismutase (FeSOD) have been reported in the SS trypanosomes than in the LS and PCF parasites (Kabiri and Steverding 2001). Actually, the LS trypanosomes with an inactive mitochondrial electron transport chain produce limited oxidants, enabling their survival even under depletion of tryparedoxin peroxidases (Bogacz et al. 2020). We conclude that the imbalance or mismatch between energy metabolism and anti-oxidant capacity in the SS trypanosomes may cause the degeneration of SS trypanosomes when they are exposed to 37 °C or higher temperatures for a long time. These oxidative stress imbalances may be a necessary evolutionary trade-off to facilitate the role of SS trypanosomes in transmission between the physiologically diverse hosts (mammals and vectors).

Inappropriate accumulation of oxidative stress within mitochondria could lead to their dysfunction. The mitochondrial membrane potential (ΔΨm) is one of the critical indicators for assessing mitochondrial function. In *T. brucei*, ΔΨm plays a pivotal role in energy generation and utilization, as well as in the transport of mitochondrial substances (Michels et al. 2021; Nolan and Voorheis 1992; Schnaufer et al. 2005; Williams et al. 2008). A decrease in ΔΨm was clearly observed in the freshly isolated SS trypanosomes, compared to the LS trypanosomes. We suggest that this decrease represents the normal mitochondrial rewiring process during the differentiation of bloodstream forms (BSFs). As is well known, SS trypanosomes lack complexes III and IV in their mitochondrial electron transport chain (Zikova 2022) and therefore the ΔΨm is likely maintained by ATP derived from glycolysis, and by the reversed function of ATP synthase (Hierro-Yap et al. 2021). Evidence indicated that a decrease in glycolysis demand, due to the arrested cell-cycle in the SS trypanosomes (Kabani et al. 2009), may contribute to the physiological decline in ΔΨm. Interestingly, even under favorable conditions, like 27 °C, the SS trypanosomes also exhibited a low ΔΨm. Previous studies have demonstrated that reducing the incubation temperature could trigger the differentiation of SS trypanosomes to the PCFs (Czichos et al. 1986). Indeed, we also observed the occurrence of PCF trypanosomes during the incubation of SS trypanosomes at 27°C. However, this was not yet accompanied by the recovery of ΔΨm once placed at 27°C. It is highly likely that solely lowering the temperature (such as to 27 °C) requires a longer duration for the SS trypanosomes to differentiate into the PCFs. Considering the mismatch of SS trypanosomes mentioned above, we believe that this low ΔΨm in the SS trypanosomes at 27 °C is also key evidence, indicating that these trypanosomes are dancing on a precarious knife-edge, that they must minimize metabolism to stop cell proliferation in order to promote a longer survival time. This status of SS trypanosomes is supported by subjecting them to temperatures above 37°C. Not only was no ΔΨm recovery detected, but also a failure to differentiate into the PCFs was observed, which included the formation of fenestrated mitochondria and driving mitochondrial membrane permeabilization. Interestingly, in *T. brucei* treated with APOL1, similar mitochondrial damage was also observed, as well as mitochondrial membrane permeabilization and apoptosis-like cell death (Vanwalleghem et al. 2015). Furthermore, the fenestration and permeabilization of mitochondrial membranes are indicative of a key event in programmed cell death (PCD), like the apoptosis pathway (Savitskaya and Onishchenko 2015). This could explain the delay of differentiation into the PCFs, following high temperature incubation (e.g. 39 °C) and the subsequent prolonging of proliferation. In this state, the stressed SS trypanosomes likely require more time to clear these impediments as mitochondrial damage likely impairs metabolic switching. The absence of ΔΨm recovery at high temperatures emphasizes the vulnerability of the parasite mitochondria and their significantly reduced ability to cope with host fever. Nevertheless, the sensitive nature of SS trypanosomes to high temperature may be a good model to investigate the changes in the transcriptomes and metabolomes of parasites which are needed to adapt to new environments when switching between vertebrate and invertebrate hosts.

Although we demonstrated that fever could induce significant metabolic disruptions in SS trypanosomes, other key reasons related to the clearance at high temperatures may also play an important role. For example, a substantial accumulation of succinate in the SS trypanosomes was detected when incubated at 39°C. Succinate, one of the end products of metabolism in trypanosomes, is usually lower than that of another end product, alanine, in the LS trypanosomes (Grant and Fulton 1957; Mazet et al. 2013). When the SS trypanosomes were maintained at 37 °C, much lower levels of succinate than alanine were found, indicating that these trypanosomes still shared the characteristic metabolic profiles with the BSFs at this temperature. However, when the SS trypanosomes were incubated at 39 °C, the succinate level dramatically increased by sixfold and accounted for close to 30% of the alanine levels, which was also increased by twofold. These phenomena are important evidence of the metabolic imbalance in these trypanosomes at 39°C. Indeed, the succinate/alanine ratio could be considered as an indicator of specific metabolic adaptations in PCFs. Studies demonstrated that the PCFs usually use proline as a carbon source in the midgut of tsetse fly and retain substantial succinate and alanine (Villafraz et al. 2021) but when glucose is used as a carbon source the PCFs retain mainly succinate but no alanine (Mazet et al. 2013). The abnormal metabolic state in the SS trypanosomes at 39 °C likely reflects restricted energy metabolism or dysfunction of related enzymes. Within trypanosomes, the production of succinate from fumarate, a reaction coupled with the transfer of electrons from NADH, is catalyzed by fumarate reductase (FRD) (Coustou et al. 2005). Previous results have shown that, compared to the LS trypanosomes, the glycosomal and mitochondrial FRD (FRDg & FRDm) are significantly expressed in the activated mitochondria of SS trypanosomes (Besteiro et al. 2002; Dejung et al. 2016). Importantly, both FRDg and FRDm have been implicated as important sources of ROS (Fang and Beattie 2002; Wargnies et al. 2021). These findings suggest that the overproduction of succinate via FRDm in mitochondria, alongside elevated ROS levels, may trigger the accelerating degeneration of SS trypanosomes during the period of host fever. Further investigation into the sources and fluxes of ROS and related metabolites among all the different stages of this parasite is crucial for uncovering the intrinsic mechanisms underlying the trypanosome metabolic adaptations to environmental changes. This could potentially lead to a better understanding of the factors driving the evolution of trypanosome species within their diverse range of vertebrate hosts, such as, fish to mammals.

Although the elimination of SS trypanosomes by host fever may occur throughout the entire infection, its role at the first parasitemia peak should be emphasized. Both the innate and adaptive immune responses are essential for pathogen clearance during infection. Evidence indicated that antigen presentation during trypanosome infections was driven by the degenerating fragments rather than the release of variant surface glycoproteins (VSGs) from the active LS trypanosomes (Black et al. 1982). Although the study explained the control of parasitaemic fluctuations during the chronic phase, it did not account for the rapid remission of the first parasitaemia which is particularly highly represented by the death of SS trypanosomes. It is generally acknowledged that IgM antibody responses, mounted after infection and detected prior to the first parasitaemic remission (~ 7 days), are responsible for clearing the first parasitaemia (Black et al. 1983, 1986; Sendashonga and Black 1982). Also, complement component 3 (C3) and the antibody-mediated phagocytosis by Kupffer cells have been identified as the remaining factors required for intravascular clearance of African trypanosomes during early infection (Liu et al. 2019; Shi et al. 2004). Under these circumstances, antibodies have been shown to be mediated for complement-mediated capture of circulating parasites by Kupffer cells. However, in our study, the D6 plasma showed no evidence of trypanocidal activity against either the LS or SS trypanosomes in vitro, suggesting that antibodies or complement may not be the decisive factors for trypanosome clearance during early infection. Importantly, some of these studies, focusing on complement and phagocytosis, were conducted with highly virulent strains of trypanosome and may have lacked consideration of trypanosome infections under natural conditions. In addition, IgM-deficient and complement C3-deficient mice were both found to be able to trigger the first parasitaemic decrease, although at a slower rate compared to the wild-type controls (Macleod et al. 2022; Magez et al. 2008). Furthermore, the IgM antibodies seemed to be dispensable for trypanosome clearance during early infection during natural infections by bites from infected tsetse flies (Magez et al. 2008). This suggests that although antibodies (mainly IgM), complement and phagocytosis together may play important roles in the remission of the first parasitaemia, they are not the sole factor involved. This means that other contributing factors need also to be considered. When considering the high parasitaemia generated from artificial infections, the natural turnover of SS trypanosomes during their differentiation may serve as the main contributing factor for the rapid clearance of the first parasitaemic peak. It is clear that our findings on the mechanisms of thermo-sensitivity of SS trypanosomes (especially when facing host fever) systematically present a very acceptable explanation for the acceleration of trypanosome clearance. It should be stressed here, however, that we tried but failed to present a fever-suppressed *T. brucei* infection model in mice. Optimistically, previous studies have already stated that the administration of potent non-steroidal anti-inflammatory drugs (NSAIDs) to animals infected with trypanosomes resulted in highly elevated parasitaemia (Dwinger et al. 1984; Zwart et al. 1990).

In the meantime, our results have confirmed that the SS trypanosomes are less sensitive to antibodies (plasma collected from mice after 21 days infected with *T. brucei*) compared to the LS trypanosomes, as well as to complement C3, as previously described (Macleod et al. 2022; McLintock et al. 1993). Some studies have found that during the later chronic stages of trypanosome infection, the SS trypanosomes but not the LS trypanosomes actually predominate within bloodstream and the infected mice may not present fever (MacGregor et al. 2011; Rijo-Ferreira et al. 2018). In our study, co-treatment of antibodies and elevated temperature resulted in the rapid elimination of SS trypanosomes within 24 h, demonstrating a higher efficiency than either antibody or elevated temperature treatment alone. This finding highlights the crucial role of fever for the host against trypanosomes during long-term chronic infections. However, although fever is less frequently observed in mouse models during chronic trypanosome infection, it remains the most common symptom in human patients, particularly since many patients are already in the chronic phase at the time of diagnosis. A particular difference is that the parasitaemia is quite low or even undetectable in human patients compared to infected mice with *T. brucei* infections. This may relate to the differences among the subspecies of *T. brucei* or the differences in the host immune systems. Interestingly, the parasitaemia also remains very low in some livestock animals, such as cattle (Doko et al. 1997; Wellde et al. 1989). Undoubtedly, these cattle infected with trypanosomes also showed febrile symptoms during the time of the first peak of parasitaemia (Wellde et al. 1989), consistent with the observations in our study. Meanwhile, some researchers have suggested this trypanotolerance phenomenon is correlated with animal breeding (Malatji 2022; Murray et al. 1990). On the one hand, these livestock animals may possess specific, yet undiscovered, anti-parasite molecules, similar to the TLFs found in human. On the other hand, it is crucial to note that the baseline body temperature of these livestock animals is significantly higher than that of humans (Piccione and Refinetti 2003). This may contribute to the suppression of the proliferation of LS trypanosomes and the accelerated clearance of SS trypanosomes, thus resulting in a low parasitaemia. Meanwhile, the plasticity of LS trypanosomes still enables these livestock animals to serve as reservoirs for transmission (Schuster et al. 2021), although less efficient than hosts with high proportions of SS trypanosomes.

Unlike these experimental infection models, natural infections, caused by tsetse bites, generally exhibit a low first parasitaemic peak (Caljon et al. 2016; Magez et al. 2008). Although we do not have precise results regarding the first parasitaemic peak in naturally infected animals or human patients, it is highly possible that it may not be the same for hosts that are experimentally and naturally infected. In natural infections, from infected tsetse flies, the metacyclic forms (MCFs) of *T. brucei* are injected into the mammalian host and they need to develop into the LS trypanosomes before they can establish a parasitaemia in the bloodstream (Reuter et al. 2023; Vickerman 1985). However, the subsequent appearance of a peak of parasitaemia indicates the initial LS parasite proliferation is followed by repression. This repression or clearance is usually attributed to an immune response. However, if the post-peak phase involves a degree of transition of the LS to SS trypanosomes, as seen in our experimental study and many other studies using pleomorphic strains, then this may require a reconsideration of the role of temperature in the trypanosome growth dynamics. Incorporation of the notion of a transition from the LS to SS trypanosomes in our understanding of parasitaemic peak formation and decline, following the natural transmission, will require the incorporation of SS trypanosome survival as a factor. In our study, we highlight the importance of temperature in accelerating the destruction of SS trypanosomes and, therefore, as a possible contributary factor to the post-peak phase. In natural infections, it is likely that the first parasitaemia does not reach similarly high levels, compared with our experimental models, and may not cause such a rapid induction of fever. However, a low level fever, occurring before the generation of specific antibodies, may still function in controlling the initial stages of parasitaemia during natural infections. Undoubtedly, it is supported by the thermosuppression of the proliferation of LS trypanosomes under 38 °C conditions. In fact, fever-related accelerated clearance may still be the main mechanism for the initial control of parasitaemia, of course based on, and occurring after, the differentiation of parasites. A number of case studies of human disease show that the fever, either high or low-grade, is one of the most common clinical signs in patients infected with *T*. *brucei* (Chen et al. 2019; Liu et al. 2018; Papagni et al. 2023; Paul et al. 2014; Shah et al. 2022; Yagnik et al. 2021). Interestingly, a recent study showed that a shift of balance from temperature-related clearance to antibody-dominated immunity occurred in the later stages of infection (Rijo-Ferreira et al. 2018). This is likely to be due to the fact that fever acts as a double-edged sword in innate immunity, necessitating tight regulation by warm-blooded hosts. Additionally, the mammalian body displays a temperature gradient from the core (heart, liver, lung, etc.) to the physical surface, where peripheral tissues can drop to as low as 30 °C (Taylor et al. 2014). Since the surprising discovery that the subcutaneous region can act as a reservoir for trypanosomes (Capewell et al. 2016), high temperature-associated clearance may shed light on the evolutionary advantages of the location of such a reservoir. Therefore, we propose that the residence of trypanosomes in skin or other tissues extends their survival by avoiding host fever (Capewell et al. 2016; Trindade et al. 2022, 2016), as well as extending their survival in mammals with a core body temperature above 38 °C, such as sheep and goats (Abdisa 2017; Piccione and Refinetti 2003). This strategy may also increase their potential for transmission, thus contributing to the spread of trypanosomiasis.

Taken together, our study provides critical insights into the mechanism underlying the thermo-sensitivity of the short stumpy (SS) form of *T. brucei* and its response to host fever during infection. We have demonstrated that fever plays a significant role in accelerating the clearance of SS trypanosomes and offers a reasonable explanation for the dramatically rapid remission of the first parasitaemic peak in a murine model. This temperature-accelerated clearance mechanism could complement the immune response, as the SS trypanosomes are less sensitive to antibody elimination. Future research should be focused on the mechanisms driving thermo-sensitivity, within different life cycle stages, and the parasite derived triggers for host fever. This may provide the potential to discover more effective strategies for preventing and controlling African human and animal trypanosomiasis.

## Methods and Materials

### Animals and parasites

Male and female C57BL/6 mice (8–12 weeks old) used in this study were purchased from the Laboratory Animal Center of Sun Yat-Sen University. The origin of the pleomorphic *T. brucei* AnTat1.1 (EATRO1125) strain used in this study has been previously described (Delauw et al. 1985). Each mouse was intraperitoneally injected with 2 × 10^3^ LS trypanosomes. The parasitaemia and the body temperature (via rectal measurement) were monitored daily. This work was conducted in accordance with protocols approved by the Laboratory Animal Use and Care Committee of Sun Yat-Sen University under the license No.32270446.

The collection of SS trypanosomes was performed as follows. At 6 dpi, mice were sacrificed to collect SS trypanosomes after DAPI staining from tail blood verified that > 99% of the parasites had differentiated. Blood was collected via cardiac puncture, and trypanosomes were purified via DEAE-52 cellulose (Whatman, UK) immediately. The proportion of LS and SS forms in the samples was confirmed again by microscopy for later experiments.

### Trypanosome bioassays

For the antibody mediated trypanocidal assay, the parasites were washed and incubated in HMI-9 medium supplemented with 10% (v/v) fetal bovine serum (FBS) (Hirumi and Hirumi 1989) or 5% (v/v) FBS plus 5% (v/v) corresponding plasma. For mouse plasma preparation, blood collected via cardiac puncture was immediately supplemented with heparin sodium, with centrifugation at 2,000 × *g* for 10 min. The plasma was collected from the supernatant and passed through a 0.22 µm filter (Merck Millipore, USA).

For the temperature sensitivity assay, trypanosomes were incubated at the designated temperature in HMI-9 medium as described above. For the procyclic form (PCF) differentiation test, trypanosomes were incubated at 27 °C in 15% (v/v) FBS/DTM medium with the addition of *cis*-Aconitic acid (Vassella and Boshart 1996) for 48 h and transferred to 10% (v/v) FBS/SDM-79 medium (Brun and Schonenberger 1979).

### Generation of PAD1:P2A:tdTomato cell line

In situ endogenous C-terminal tdTomato tagging for PAD1 was performed using the pEnT6 (neomycin) vector (Kelly et al. 2007), incorporating a P2A sequence (Kim et al. 2011) between PAD1 and tdTomato. Plasmids, linearized by *Xho* I, were transfected into trypanosomes by electroporation. The transfected cells were selected by the addition of 3 μg/mL G418 in HMI-9 medium containing 10% (v/v) FBS. Primers used are listed in Table S3.

### Analysis of ROS and monitoring mitochondrial membrane potential ΔΨm

For the ROS assay and membrane potential monitoring, cells were incubated with MitoSOX Green (Thermo Fisher Scientific, USA) and Rhodamine 123 (Sigma-Aldrich, USA), respectively. After incubation at room temperature for 30 min, cells were centrifuged at 1,000 × *g* for 10 min and re-suspended in PBS. Fluorescent signals were either acquired by flow cytometry (Beckman CytoFLEX, USA) and analyzed by FlowJo (Version 10.8.1), or by fluorescence microscopy (Leica DM4B, Germany).

### Fluorescence microscopy

Mitochondria were stained with MitoTracker Red (Invitrogen, USA) for 20 min in HMI-9 medium and cells were then harvested and washed before preparation of wet mounts for imaging. For imaging of nuclei, slides were fixed with methanol and stained with DAPI (40 µg/mL) for 15 min. Images were captured by using a Leica DM4B microscope and analyzed with ImageJ software (Version 2.14.0/1.54f).

### Trypanosome phosphatidylserine exposure assay

The detection of apoptosis-like cell death via phosphatidylserine exposure in trypanosomes was carried out as described in previous studies (Banerjee et al. 2017; Levy et al. 2015; Lim et al. 2019; Rani et al. 2021). Briefly, harvested trypanosomes were washed and incubated in Annexin V/PI staining solution (Beyotime, China) according to the manufacturer’s instructions. After incubation for 20 min at room temperature in the dark, cells were washed three times and were assayed by flow cytometry (Beckman CytoFLEX, USA) as soon as possible. Data were analyzed by FlowJo software (Version 10.8.1).

### Detection of anti-trypanosome IgG, IgM and complement C3 in plasma from infected mice

For the detection of anti-trypanosome IgG, IgM and complement C3 in plasma from infected mice, freshly collected LS and SS trypanosomes were washed and separated by SDS-PAGE and transferred onto a nitrocellulose membrane. The membrane was blocked with 5% non-fat milk for 4 h, followed by incubation with 1:1000 (v/v) diluted mouse plasma or FBS as the primary antibody in TTBS buffer (10 mM Tris–HCl, 150 mM NaCl, 0.1% (v/v) Tween-20) overnight at 4°C. After washing with TTBS, anti-mouse IgG(H + L) antibody (Thermo Fisher Scientific, USA), anti-mouse IgM(H + L) antibody (BOSTER, China) and anti-C3 antibody (proteintech, China) were diluted 1:5000 (v/v) and applied for 45 min at room temperature.

For the detection of parasite-bounded IgG, IgM and complement C3 on the LS and SS trypanosomes, trypanosomes co-cultured with D0 plasma, D6 plasma and D21 plasma for 24 h were collected and washed at least three times, then separated and transferred onto a nitrocellulose membrane as above. The antibodies for IgG, IgM and complement C3 were the same as those mentioned above.

The loading control was confirmed with the HRP-conjugated beta tubulin antibody (proteintech, China). The membrane was washed and developed using ECL reagents (Tanon, China), and signals were captured by using an imaging system (Tanon 5200 Chemiluminescence Imaging System, China).

### RNA extraction and reverse transcription quantitative PCR (RT-qPCR)

Total RNA was extracted from cell samples using a classical protocol involving Trizol reagent (Invitrogen, USA) and chloroform. RNA integrity and concentration were assessed using a NanoDrop 2000 C spectrophotometer (Thermo Fisher Scientific, USA). cDNA synthesis was performed using the HiScript III RT SuperMix for qPCR (+ gDNA wiper) Kit (Vazyme, China) with 1 µg of total RNA as the template according to the manufacturer's instructions. Quantitative PCR was performed using the ChamQ Universal SYBR qPCR Master Mix Kit (Vazyme, China) on a LightCycler480 (Roche, Switzerland). The qPCR amplification conditions were as follows: an initial denaturation at 95 °C for 5 min, followed by 45 cycles of denaturation at 95 °C for 10 s, annealing at 60 °C for 20 s and extension at 72 °C for 20 s. Gene expression levels were assessed using the 2^−ΔΔCt^ method and normalized to the heat-insensitive gene *28S rRNA* as previously described (Ooi et al. 2020). The experiments were conducted in three independent biological replicates. Each sample was run in triplicate, and the average Ct values were used for the calculations. Statistical significance was assessed using a two-tailed paired Student’s *t*-test for transcripts with ≥ twofold change. Primers used are listed in Table S4.

### Ultrastructural analysis by transmission electron microscopy

SS trypanosomes were harvested and washed with PBS by centrifugation at 1,000 *g* for 10 min. Samples were fixed with cold 2.5%/2% (v/v) glutaraldehyde/paraformaldehyde in PBS solution overnight at 4°C. For transmission electron microscopy, trypanosomes were post-fixed with 1% osmium tetroxide (OsO_4_) for 1 h and then washed in distilled water and dehydrated through a graded ethanol series (30%, 50%, 70%, 90%, 100%). The samples were then infiltrated and embedded in resin overnight and left to polymerize for 2 days at 60°C. Ultra-thin Sects. (100 nm) were cut and collected on copper grids. The stained sections were examined with a transmission electron microscope (JEOL JEM-1400FLASH, Japan).

### Metabolomic analysis

Samples from six biological replicates were collected and snap-frozen in liquid nitrogen and stored at − 80 °C until further processing. All the samples were thawed on ice and mixed with 1 ml of cold (2:2:1, v/v/v) methanol/acetonitrile/ddH_2_O. The homogenates were sonicated at 4 °C for 30 min for twice and centrifuged at 4°C. The supernatants were collected and dried under vacuum. For LC–MS analysis, all the samples were re-dissolved in 100 μl (1:1, v/v) acetonitrile/ddH_2_O solvent and analyzed using a mass spectrometer (AB SCIEX Triple TOF 6600, USA), with metabolites detected in both positive and negative ion modes. To identify and visualize differences in metabolite profiles, unsupervised principal component analysis (PCA) was employed for dimensionality reduction, while orthogonal projections to latent structures-discriminant analysis (OPLS-DA) was used to determine statistically significant differences between the two temperature conditions. A variable importance in projection (VIP) score derived from the OPLS-DA model was used to rank metabolites based on their contribution to group separation. Metabolites with a fold change > 2 or < 0.5, a *p*-value from the *t*-test < 0.05 and a VIP score ≥ 1 were considered significantly different between the two groups. Molecular structures of metabolites were identified by matching their mass-to-charge ratios (m/z) and retention times to the Human Metabolome Database (HMDB) or MassBank NIST database. The schematic diagrams of the metabolic pathways in this study (Fig. 7b; Fig. S8e) were conducted based on a canonical model in a previous study (Michels et al. 2021).

### Statistical analysis

All statistical analyses were performed using GraphPad Prism software (Version 10.2.1).

Correlations between parasitaemia and body temperature were assessed using the Pearson correlation coefficient. The multiple linear regression model using the least squares method was applied to evaluate the relationship between the dependent variable (Day567_P and Day567_SS) and five time-point predictors (Day123_T, Day234_T, Day345_T, Day456 _T and Day567_T). Residual normality was confirmed via Shapiro–Wilk and Anderson–Darling tests, and multicollinearity was assessed using variance inflation factors (VIFs < 5).

For multiple comparisons in Fig. 2a, data were analyzed by two-way ANOVA with the two factors defined as temperature and time. Post-hoc pairwise comparisons were conducted to evaluate differences among groups at each time point. To control for multiple testing, raw *p*-values obtained from the multiple comparisons were adjusted using the original false discovery rate (FDR) procedure of Benjamini and Hochberg. Adjusted *p*-values < 0.05 were considered statistically significant, and groups that do not share the same lowercase letter are significantly different after Benjamini–Hochberg correction. Exact *p*-values and adjusted *p*-values are reported in Table S5.

Statistical significance was assessed using either a two-tailed Student’s *t*-test or a two-tailed Welch's *t*-test based on an *F*-test for homogeneity of variances (Welch's *t*-test was applied if the *p* value of the *F*-test was less than 0.05). Significance levels were denoted as follows: ns *p* ≥ 0.05, * *p* < 0.05, ** *p* < 0.01, *** *p* < 0.001, **** *p* < 0.0001.

Image analysis was conducted using ImageJ software (Version 2.14.0/1.54f).

## Supplementary Information

Supplementary Material 1.

## Data Availability

The data and materials that support the findings of this study are not openly available due to reasons of sensitivity and are available from the corresponding author upon reasonable request.
