# Supplementary material for: Thermo-related degeneration of stumpy forms of Trypanosoma brucei, the pathogen of African sleeping sickness
Source: Adv Biotechnol (Singap). 2025 Sep 23;3(4):28. doi: 10.1007/s44307-025-00081-9 (PMC12457267; doi:10.1007/s44307-025-00081-9)
Supplement: Supplementary file 1 — Supplementary Material 1. [file 44307_2025_81_MOESM1_ESM.pdf]

# Supplementary Information (SI)
# Advanced Biotechnology

# Thermo-related degeneration of stumpy forms of *Trypanosoma brucei*, the pathogen of African sleeping sickness

Jia-Yi Luo[a], Ju-Feng Wang[a], Jiong Yang[a], Peng Zhang[a,*], Geoff Hide[b], De-Hua Lai[a,#], Zhao-Rong Lun[a,b]

[a] MOE Key Laboratory of Gene Function and Regulation, State Key Laboratory of Biocontrol and Guangdong Provincial Key Laboratory of Aquatic Economic Animals, School of Life Sciences, Sun Yat-Sen University, Guangzhou 510275, The People's Republic of China.

[b] Biomedical Research and Innovation Centre, School of Science, Engineering and Environment, University of Salford, Salford M5 4WT, UK.

[*] Present address. Southern Marine Science and Engineering Guangdong Laboratory (Guangzhou), Guangzhou 511458, The People's Republic of China.

[#] Corresponding author. E-mail address: laidehua@mail.sysu.edu.cn (De-Hua Lai).

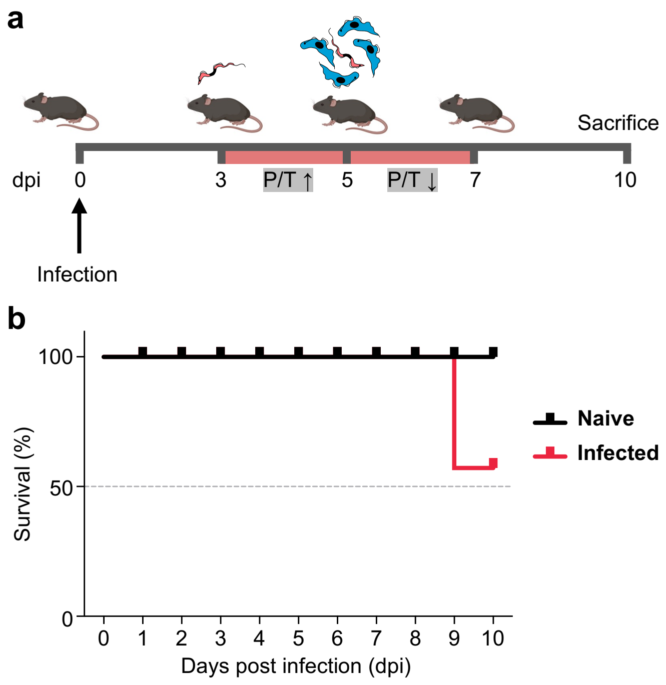


**Supplementary Fig. 1** Workflow of mice trypanosome infections. **a** Diagram of trypanosome infection procedures. Mice (n = 7) were intraperitoneally (i.p.) infected with 2 × $10^3$ LS trypanosomes. The parasitaemia and the body temperature were monitored daily. Abbreviations, P for parasitaemia, T for body temperature. The trypanosome diagrams indicate the presence of LS trypanosomes (red) and SS trypanosomes (blue). **b** The survival curve tracks the survival of mice over the 10 days post-infection with *T. brucei*. The naive group consisted of non-infected mice, while the infected group was subjected to intraperitoneal infection with *T. brucei* AnTat1.1 strain. Both groups (4 naive and 7 infected mice) were monitored daily for mortality signs.

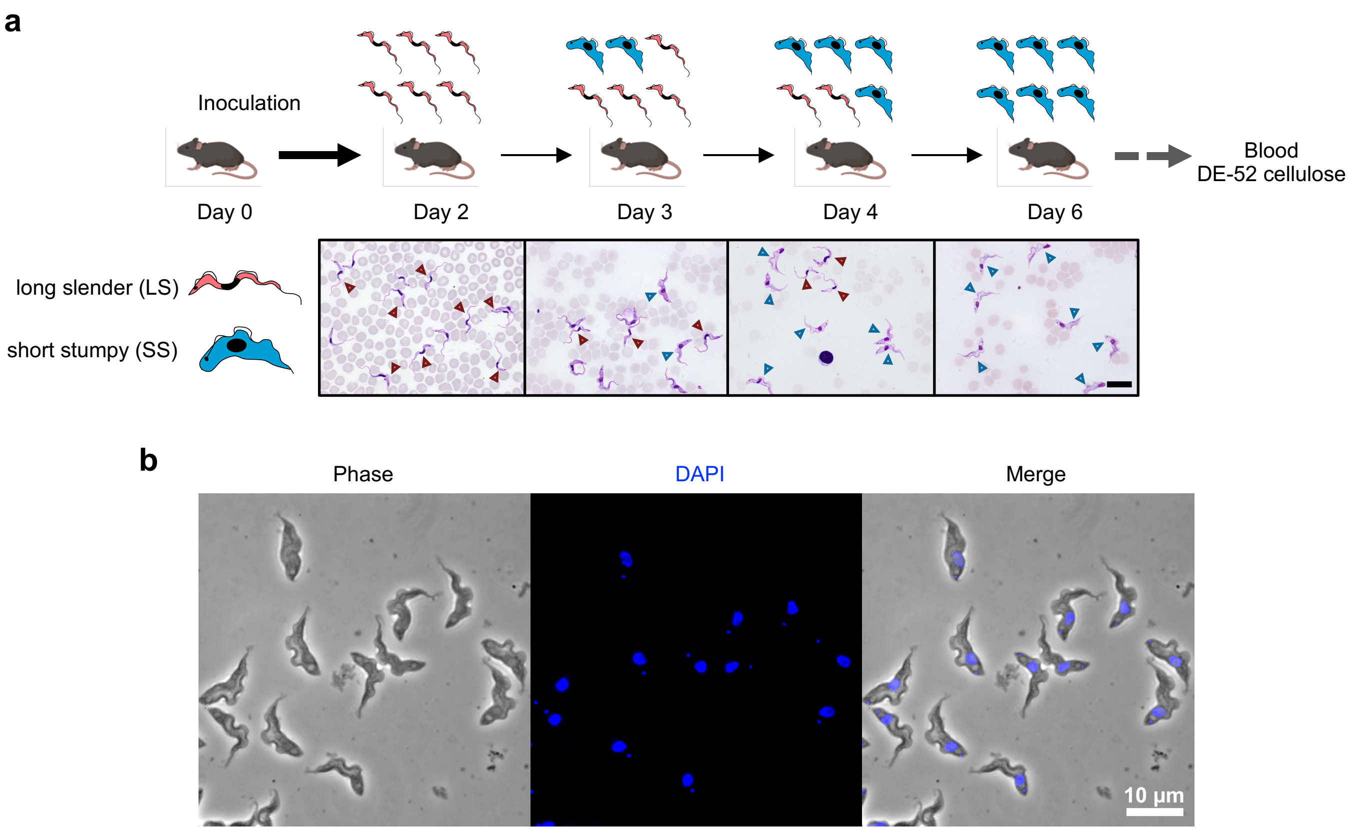


**Supplementary Fig. 2** Isolation of short stumpy trypanosomes. **a** A diagram of the SS trypanosome collection from infected mice. Blood from infected mice at 6 dpi were collected by cardiac puncture and parasites were immediately isolated and purified using DE-52 cellulose. The trypanosome diagrams indicate the presence of LS trypanosomes (red) and SS trypanosomes (blue). **b** Phase, DAPI and merged images highlighting the kinetoplast DNA and nuclear in the SS trypanosomes. Each examination used at least 300 trypanosomes to confirm the purity of these parasites (>99%).

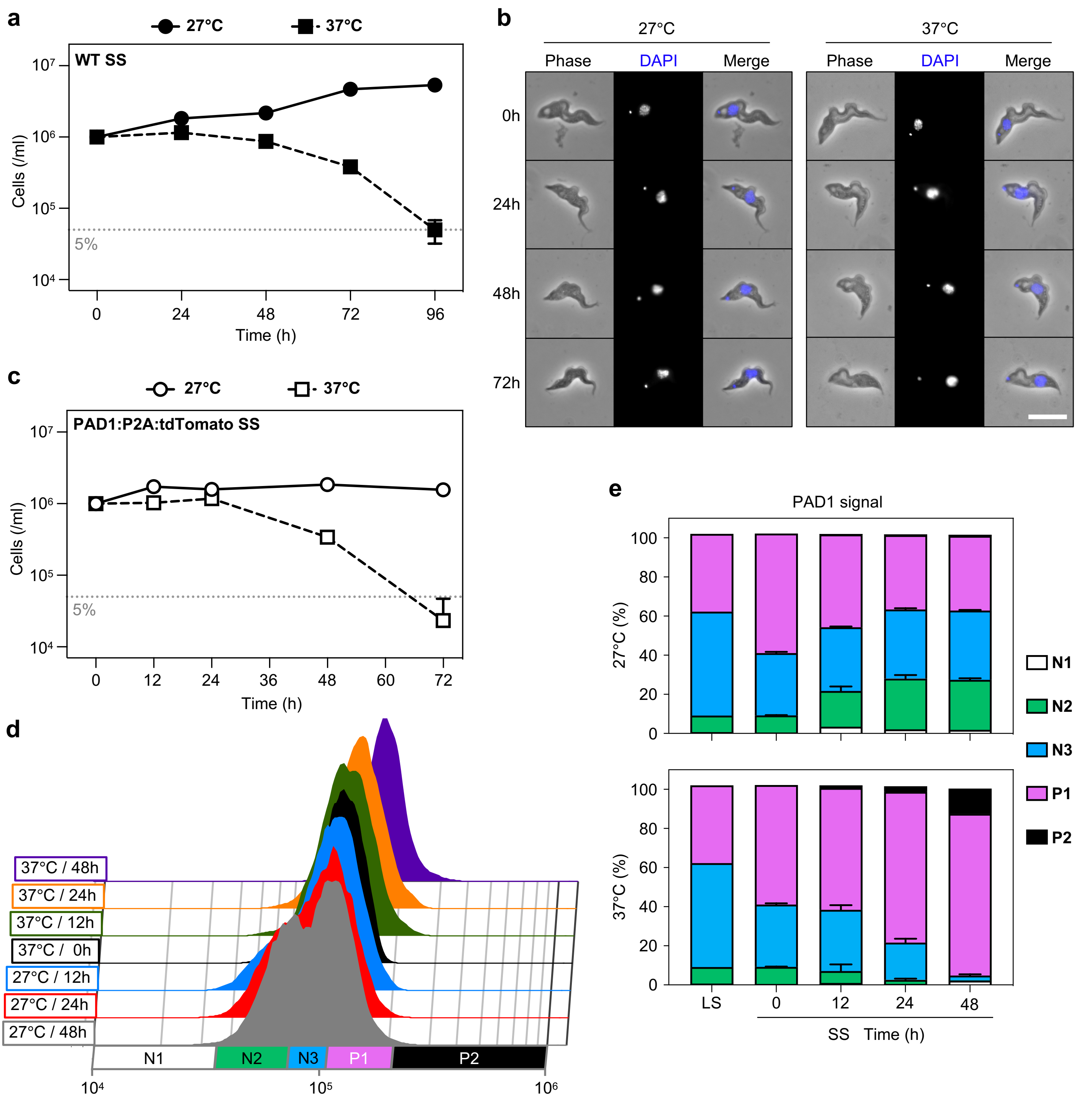


**Supplementary Fig. 3** The sensitivity of short stumpy trypanosomes to different temperatures. **a** The status of SS trypanosomes at 27°C and 37°C both in HMI-9 medium during the period over 96 hours. **b** The morphological analysis of SS trypanosomes incubated at 27°C and 37°C. Bar, 10 μm. **c** The status of SS trypanosomes of the PAD1:P2A:tdTomato cell line at 27°C and 37°C both in HMI-9 medium during the period of 72 hours. **d**, **e** The expression and distribution of PAD1 in the SS trypanosomes with five intensity gates along different incubation times by flow analysis in HMI-9 medium. For flow gating, the expression of PAD1 was divided into three negative gates (N1, the unlabeled WT trypanosomes; N2, the cells with almost no signal; N3, the typical LS trypanosomes of the cell line) and two positive gates (P1, the typical SS trypanosomes of the cell line; P2, the cells with high PAD1 signal). Results are shown as a mean ± SD.

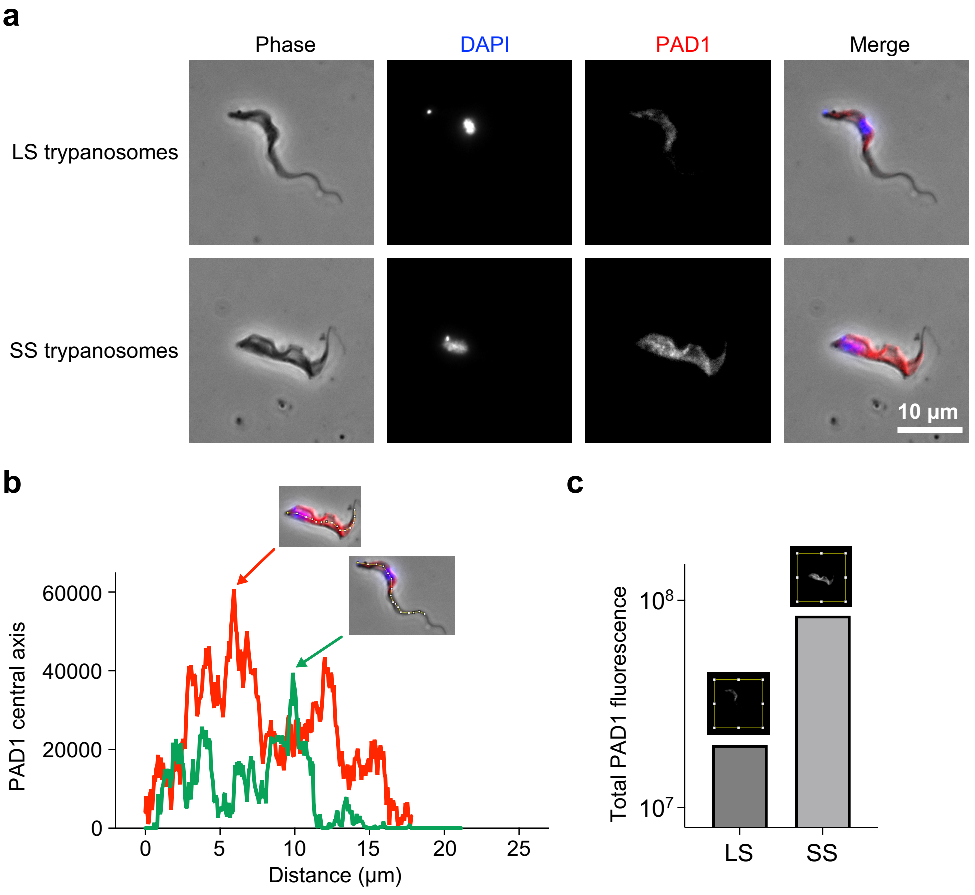


**Supplementary Fig. 4** Immunofluorescence imaging of the PAD1:P2A:tdTomato cell line. **a** Phase, DAPI, PAD1 and merged images of PAD1:P2A:tdTomato cell line for LS and SS trypanosomes. **b** Fluorescence intensity profiles of PAD1 along the longitudinal axis of the parasites for both forms. **c** Bar graph showing the quantitative analysis of total PAD1 fluorescence intensity in these two forms. Image analyses were performed via ImageJ.

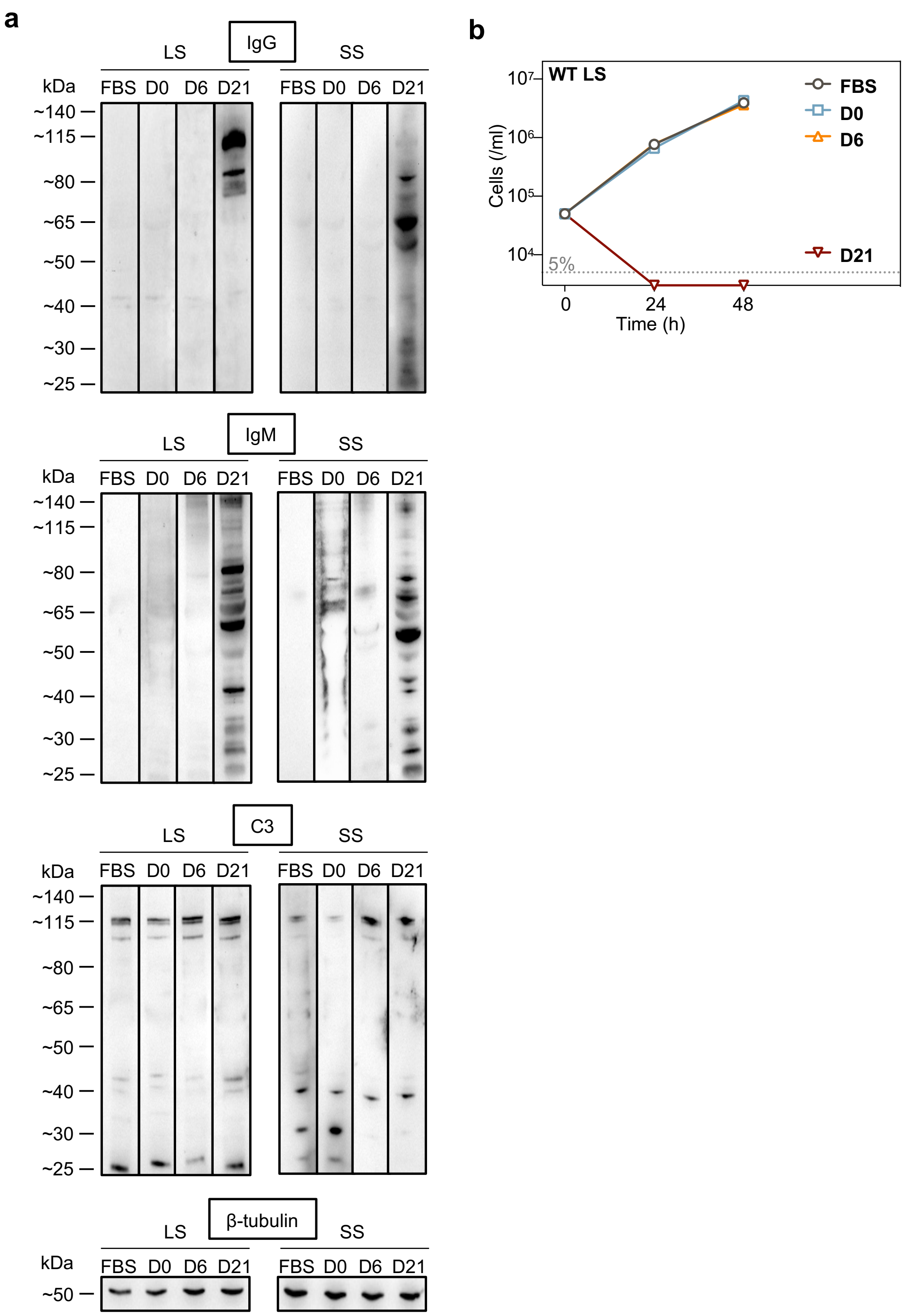


**Supplementary Fig.5** Validation of anti-trypanosome antibodies and complement C3 in plasma from trypanosome infected mice. **a** Confirmation of anti-trypanosome IgG in D21 plasma, IgM in both D6 and D21 plasma, C3 in FBS and all the D0, D6 and D21 plasma. Samples equivalent to $5×10^5$ cells were taken from the freshly isolated LS and SS trypanosomes and loaded in each lane via anti β-tubulin confirmation, with the addition of plasma from mice as the primary antibody and the anti mouse IgG(H+L), anti mouse IgM(H+L) and anti C3 as secondary antibody for antibodies and complement detection via enhanced chemiluminescence. **b** The effects of anti-trypanosome abilities of mice plasma on the LS trypanosomes at 37°C in HMI-9 medium. Results are shown as a mean ± SD.

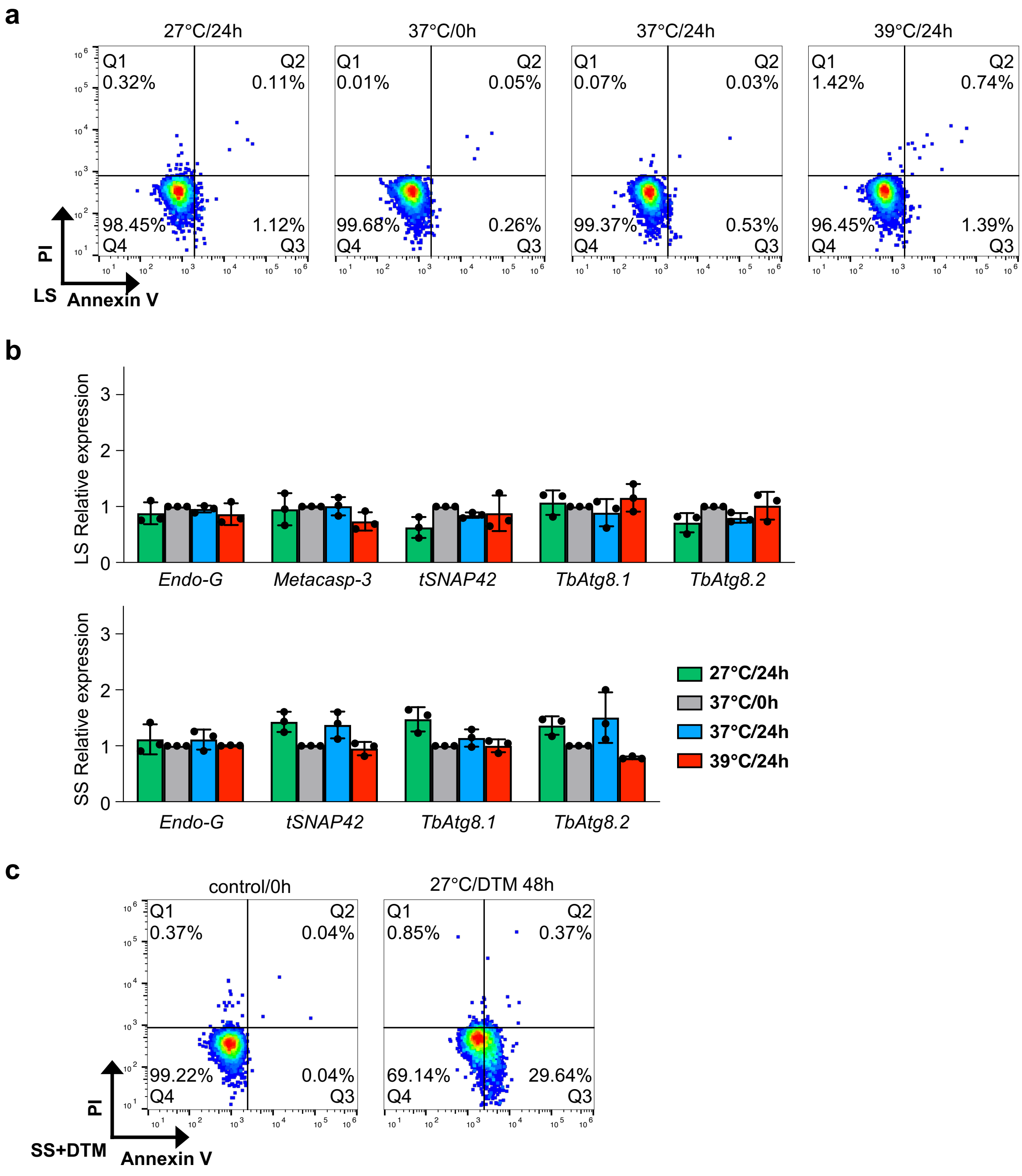


**Supplementary Fig. 6** Evaluation of apoptosis-like events in long slender trypanosomes at the designated temperatures and SS trypanosome after DTM induction. **a** Analysis of phosphatidylserine exposure in the LS trypanosomes after 24-hour culture by flow cytometry at different temperatures in HMI-9 medium. **b** Relative expression levels of the apoptosis-like associated genes and the autophagy-related genes in the LS and SS trypanosomes at different temperatures in HMI-9 medium using RT-qPCR. The data are normalized to the reference gene *28S rRNA*. Results are shown as a mean ± SD. The experiments were conducted in three independent biological replicates. **c** Analysis of phosphatidylserine exposure in the SS trypanosomes after 48-hour DTM induction at 27°C by flow cytometry.

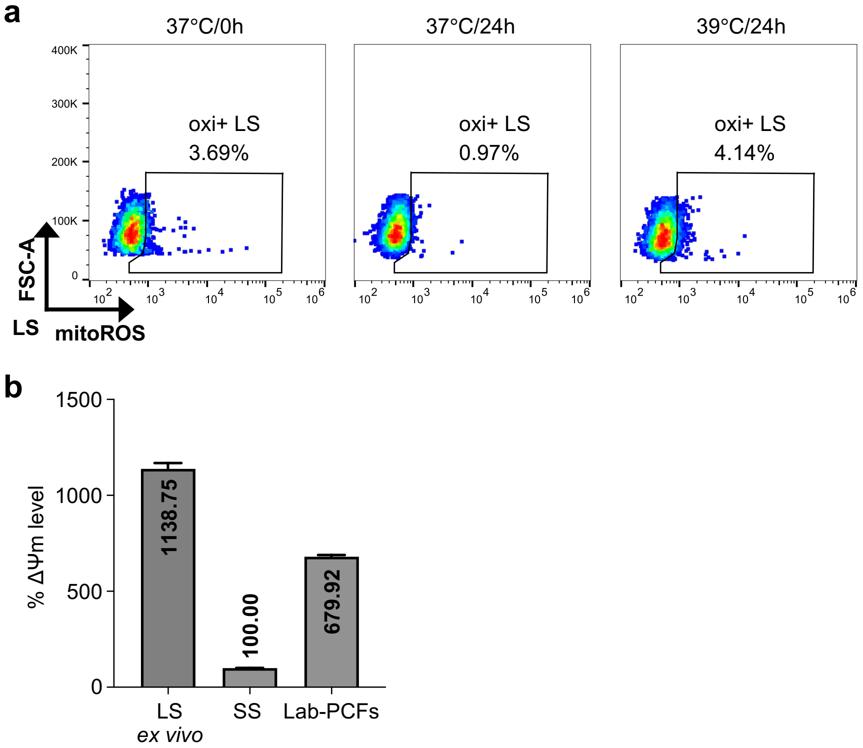


**Supplementary Fig. 7** Evaluation of oxidative stress in long slender trypanosomes and the levels of mitochondrial membrane potential of *T. brucei*. **a** Flow cytometry fluorescence histograms of mitochondria ROS levels in the LS trypanosomes after 24-hour incubation at 37°C and 39°C in HMI-9 medium. The gating of oxi+ LS refers to those trypanosomes with oxidative stress. **b** Comparative analysis of mitochondrial membrane potential (ΔΨm) levels in freshly isolated LS and SS trypanosomes, and lab-adpated procyclic trypanosomes. Results are shown as a mean ± SD.

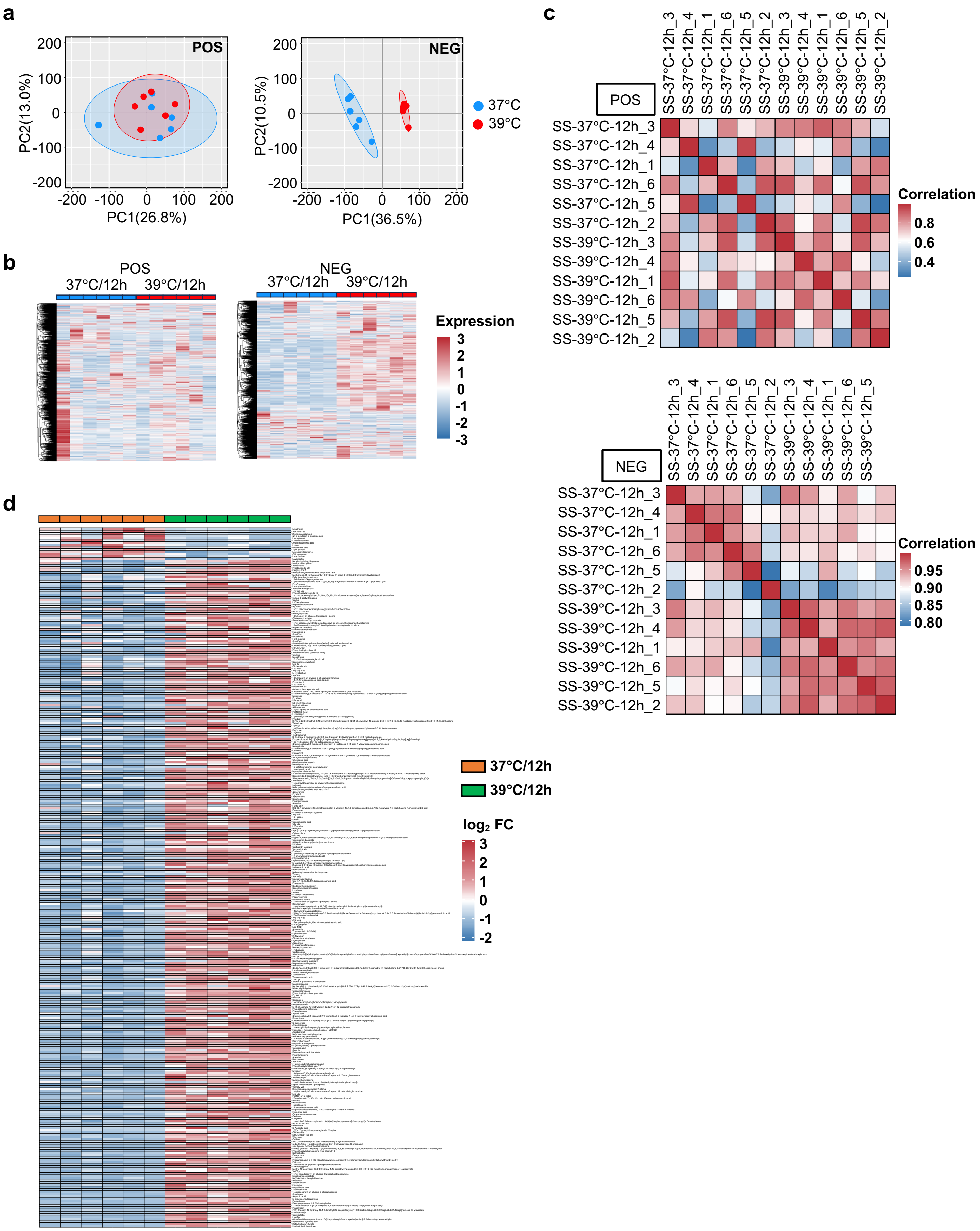
a
POS
PC2(13.0%)
PC1(26.8%)
NEG
PC2(10.5%)
PC1(36.5%)
200
100
0
-100
-200
37°C
39°C
b
POS
37°C/12h
39°C/12h
NEG
37°C/12h
39°C/12h
Expression
3
2
1
0
-1
-2
-3
c
POS
SS-37°C-12h_3
SS-37°C-12h_4
SS-37°C-12h_1
SS-37°C-12h_6
SS-37°C-12h_5
SS-37°C-12h_2
SS-39°C-12h_3
SS-39°C-12h_4
SS-39°C-12h_1
SS-39°C-12h_6
SS-39°C-12h_5
SS-39°C-12h_2
Correlation
0.8
0.6
0.4
NEG
Correlation
0.95
0.90
0.85
0.80
d
37°C/12h
39°C/12h
log2 FC
3
2
1
0
-1
-2

e

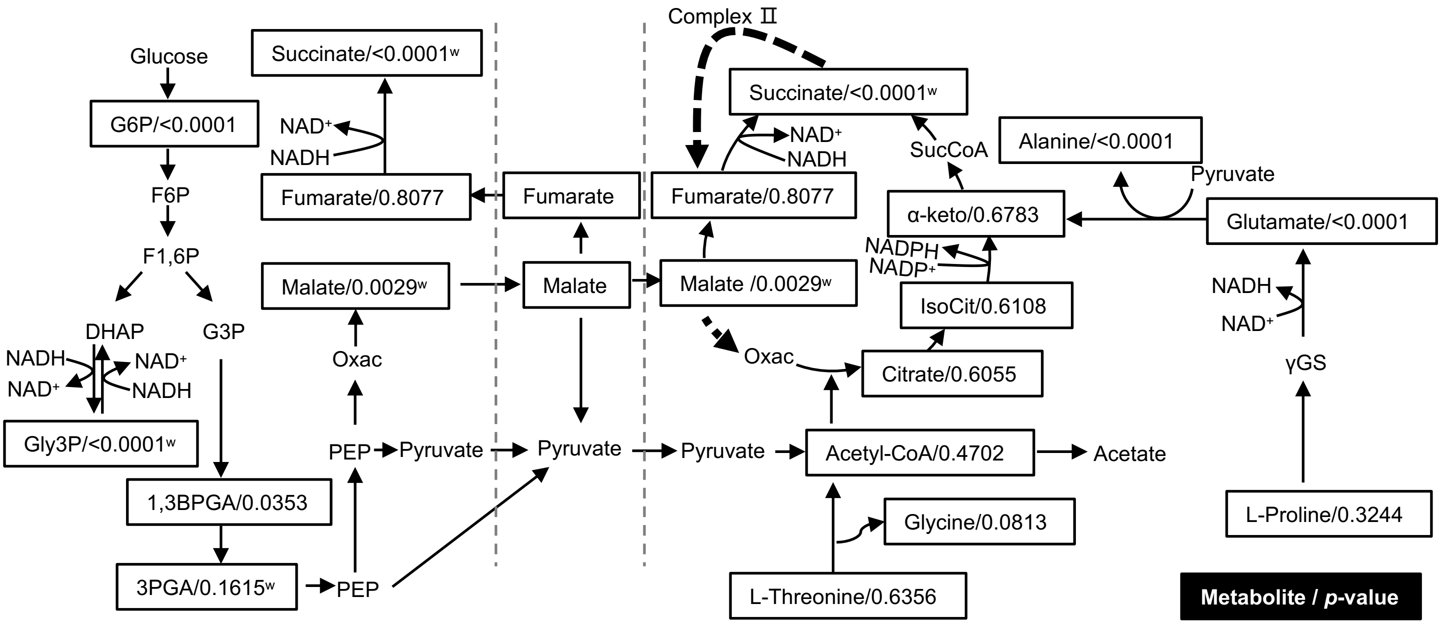


f

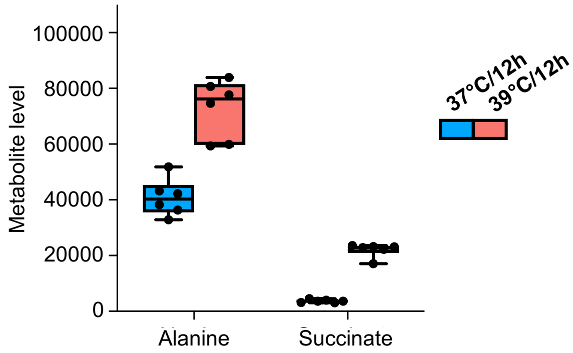


**Supplementary Fig. 8** Metabolomic profiling of short stumpy trypanosomes incubated at 37°C and 39°C for 12 hours. **a** Principal component analysis (PCA) plots of untargeted metabolomics data from the SS trypanosomes incubated at 37°C (blue) and 39°C (red) for 12 hours in HMI-9 medium, detected in positive (POS) and negative (NEG) ionization modes. **b** Heatmap of all detected metabolites in POS and NEG ionization modes. Each row represents an individual metabolite, with the color scale indicating relative abundance (log2 fold change) between the two temperatures. **c** Correlation heatmaps depicting the consistency and clustering of metabolic profiles under two temperatures in the SS trypanosomes. Heatmaps were generated for both POS mode and NEG mode data. **d** Heatmap showing the relative abundance of the 306 differential metabolites. Each row represents an individual metabolite, with the color scale indicating relative abundance ($log_2$ fold change) across the two temperatures. **e** The *t* test *p*-values showing fold changes of selected metabolites in the SS trypanosomes at 39°C compared to 37°C. G6P, glucose-6-phosphate. F6P, fructose-6-phosphate. F1,6P, fructose-1,6-biphosphate. DHAP, dihydroxyacetone phosphate. Gly3P, glycerol-3-phosphate. G3P, glyceraldehyde-3-phosphate. 1,3BPGA, 1,3-bisphosphoglycerate. 3PGA, 3-phosphoglycerate. PEP, phosphoenolpyruvate. Oxac, oxaloacetate. IsoCit, isocitrate. α-keto, α-ketoglutarate. γGS, γ-glutamate semialdehyde. The *p* values of significant differences were performed using a two-tailed unpaired Student's *t*-test or a two-tailed unpaired Welch's *t*-test (indicated by the superscript 'w' letter after the value). **f** Metabolites of alanine and succinate expression in the SS trypanosomes at 37°C and 39°C.

**Supplementary Table 1** Multiple linear regression analysis between body temperatures and parasitaemia levels.

Sheet 1/3

| Day567 P↓ | Day567 SS↓ | Day123 T | Day234 T | Day345 T↑ | Day456 T | Day567 T↓ |
|---|---|---|---|---|---|---|
| 245000000 | 184408602 | 37.1 | 36.8 | 36.6 | 38.4 | 38 |
| 240000000 | 199245283 | 36.3 | 37 | 36.9 | 37.2 | 38.1 |
| 325000000 | 238192182 | 36.6 | 36.6 | 37.1 | 38 | 38.6 |
| 390000000 | 265603448 | 36.3 | 37.1 | 37.2 | 38 | 37.9 |
| 230000000 | 185662651 | 36.8 | 37.1 | 37.1 | 37.8 | 38 |
| 330000000 | 209820896 | 37 | 36.4 | 37.7 | 38 | 38 |
| 470000000 | 346012270 | 36.7 | 36.8 | 36.9 | 38 | 37.9 |
| 120000000 | 118157895 | 36.8 | 36.6 | 38.4 | 38 | 36.9 |
| 0 | 0 | 37 | 36.9 | 37.2 | 38.1 | 36.5 |
| 35000000 | 34500000 | 36.6 | 37.1 | 38 | 38.6 | 37.3 |
| 45000000 | 42018927.4 | 37.1 | 37.2 | 38 | 37.9 | 36.9 |
| 3000000 | 2953125 | 37.1 | 37.1 | 37.8 | 38 | 36.9 |
| 60000000 | 58120104.4 | 36.4 | 37.7 | 38 | 38 | 36.9 |
| 180000000 | 176118598 | 36.8 | 36.9 | 38 | 37.9 | 36.9 |
| 0 | 0 | 36.6 | 38.4 | 38 | 36.9 | 36.5 |
| 0 | 0 | 36.9 | 37.2 | 38.1 | 36.5 | 36.8 |
| 0 | 0 | 37.1 | 38 | 38.6 | 37.3 | 36.1 |
| 0 | 0 | 37.2 | 38 | 37.9 | 36.9 | 33.8 |
| 0 | 0 | 37.1 | 37.8 | 38 | 36.9 | 36.9 |
| 0 | 0 | 37.7 | 38 | 38 | 36.9 | 35.5 |
| 0 | 0 | 36.9 | 38 | 37.9 | 36.9 | 34.3 |

**Supplementary Table 1** Multiple linear regression analysis between body temperatures and parasitaemia levels. Sheet 2/3

| Table Analyzed | |
|---|---|
| Dependent variable | Day567 P↓ |
| Regression type | Least squares |

Model

| Analysis of Variance | SS | DF | MS | F (DFn, DFd) | P value |
|---|---|---|---|---|---|
| Regression | 3.184E+17 | 5 | 6.369E+16 | F (5, 15) = 6.242 | P=0.0025 |
| Day123 T | 9.978E+15 | 1 | 9.978E+15 | F (1, 15) = 0.9780 | P=0.3384 |
| Day234 T | 1.687E+16 | 1 | 1.687E+16 | F (1, 15) = 1.653 | P=0.2180 |
| Day345 T↑ | 4.845E+16 | 1 | 4.845E+16 | F (1, 15) = 4.749 | P=0.0457 |
| Day456 T | 1.236E+15 | 1 | 1.236E+15 | F (1, 15) = 0.1211 | P=0.7326 |
| Day567 T↓ | 7.441E+15 | 1 | 7.441E+15 | F (1, 15) = 0.7293 | P=0.4065 |
| Residual | 1.53E+17 | 15 | 1.02E+16 | | |
| Total | 4.715E+17 | 20 | | | |

| Parameter estimates | Variable | Estimate | Standard error | 95% CI (asymptotic) | \|t\| | P value | P value summary |
|---|---|---|---|---|---|---|---|
| β0 | Intercept | 10118818645 | 6125309738 | -2936970012 to 23174607302 | 1.652 | 0.1193 | ns |
| β1 | Day123 T | -79066241 | 79952128 | -249480168 to 91347687 | 0.9889 | 0.3384 | ns |
| β2 | Day234 T | -86561423 | 67324451 | -230060093 to 56937247 | 1.286 | 0.218 | ns |
| β3 | Day345 T↑ | -110319392 | 50622838 | -218219417 to -2419367 | 2.179 | 0.0457 | * |
| β4 | Day456 T | -18481150 | 53097099 | -131654937 to 94692637 | 0.3481 | 0.7326 | ns |
| β5 | Day567 T↓ | 27165590 | 31809809 | -40635414 to 94966593 | 0.854 | 0.4065 | ns |

| Goodness of Fit | |
|---|---|
| Degrees of Freedom | 15 |
| R squared | 0.6754 |

| Multicollinearity | Variable | VIF | $R^2$ with other variables |
|---|---|---|---|
| β0 | Intercept | | |
| β1 | Day123 T | 1.397 | 0.2841 |
| β2 | Day234 T | 2.862 | 0.6506 |
| β3 | Day345 T↑ | 1.478 | 0.3236 |
| β4 | Day456 T | 1.968 | 0.4919 |
| β5 | Day567 T↓ | 2.917 | 0.6572 |

| Normality of Residuals | Statistics | P value | Passed normality test (alpha=0.05)? | P value summary |
|---|---|---|---|---|
| D'Agostino-Pearson omnibus (K2) | 0.1009 | 0.9508 | Yes | ns |
| Anderson-Darling (A2*) | 0.1624 | 0.9349 | Yes | ns |
| Shapiro-Wilk (W) | 0.9878 | 0.9926 | Yes | ns |
| Kolmogorov-Smirnov (distance) | 0.07918 | >0.1000 | Yes | ns |

| Data summary | |
|---|---|
| Rows in table | 21 |
| Rows skipped (missing data) | 0 |
| Rows analyzed (# cases) | 21 |
| Number of parameter estimates | 6 |
| #cases/#parameters | 3.5 |

**Supplementary Table 1** Multiple linear regression analysis between body temperatures and parasitaemia levels.

Sheet 3/3

| Table Analyzed | |
|---|---|
| Dependent variable | Day567 SS↓ |
| Regression type | Least squares |

Model

| Analysis of Variance | SS | DF | MS | F (DFn, DFd) | P value |
|---|---|---|---|---|---|
| Regression | 1.716E+17 | 5 | 3.433E+16 | F (5, 15) = 6.717 | P=0.0018 |
| Day123 T | 7.796E+15 | 1 | 7.796E+15 | F (1, 15) = 1.525 | P=0.2358 |
| Day234 T | 1.076E+16 | 1 | 1.076E+16 | F (1, 15) = 2.105 | P=0.1674 |
| Day345 T↑ | 2.218E+16 | 1 | 2.218E+16 | F (1, 15) = 4.339 | P=0.0548 |
| Day456 T | 5.008E+14 | 1 | 5.008E+14 | F (1, 15) = 0.09798 | P=0.7586 |
| Day567 T↓ | 3.078E+15 | 1 | 3.078E+15 | F (1, 15) = 0.6022 | P=0.4498 |
| Residual | 7.666E+16 | 15 | 5.111E+15 | | |
| Total | 2.483E+17 | 20 | | | |

| Parameter estimates | Variable | Estimate | Standard error | 95% CI (asymptotic) | \|t\| | P value | P value summary |
|---|---|---|---|---|---|---|---|
| β0 | Intercept | 7861622828 | 4335163753 | -1378559982 to 17101805639 | 1.813 | 0.0898 | ns |
| β1 | Day123 T | -69888886 | 56585803 | -190498670 to 50720897 | 1.235 | 0.2358 | ns |
| β2 | Day234 T | -69129878 | 47648614 | -170690494 to 32430739 | 1.451 | 0.1674 | ns |
| β3 | Day345 T↑ | -74631931 | 35828113 | -150997747 to 1733885 | 2.083 | 0.0548 | ns |
| β4 | Day456 T | -11763193 | 37579262 | -91861493 to 68335107 | 0.313 | 0.7586 | ns |
| β5 | Day567 T↓ | 17470843 | 22513267 | -30515049 to 65456735 | 0.776 | 0.4498 | ns |

| Goodness of Fit | |
|---|---|
| Degrees of Freedom | 15 |
| R squared | 0.6912 |

| Multicollinearity | Variable | VIF | $R^2$ with other variables |
|---|---|---|---|
| β0 | Intercept | | |
| β1 | Day123 T | 1.397 | 0.2841 |
| β2 | Day234 T | 2.862 | 0.6506 |
| β3 | Day345 T↑ | 1.478 | 0.3236 |
| β4 | Day456 T | 1.968 | 0.4919 |
| β5 | Day567 T↓ | 2.917 | 0.6572 |

| Normality of Residuals | Statistics | P value | Passed normality test (alpha=0.05)? | P value summary |
|---|---|---|---|---|
| D'Agostino-Pearson omnibus (K2) | 0.449 | 0.7989 | Yes | ns |
| Anderson-Darling (A2*) | 0.1778 | 0.9075 | Yes | ns |
| Shapiro-Wilk (W) | 0.987 | 0.9895 | Yes | ns |
| Kolmogorov-Smirnov (distance) | 0.08143 | >0.1000 | Yes | ns |

| Data summary | |
|---|---|
| Rows in table | 21 |
| Rows skipped (missing data) | 0 |
| Rows analyzed (# cases) | 21 |
| Number of parameter estimates | 6 |
| #cases/#parameters | 3.5 |

**Supplementary Table 2** Selected metabolites in glycolysis and the TCA cycle in SS trypanosomes incubated at different temperatures for 12 hours.

| Alignment ID | Average Rt (min) | Average Mz | Metabolite name | stumpy-37-12h-1 | stumpy-37-12h-2 | stumpy-37-12h-3 | stumpy-37-12h-4 | stumpy-37-12h-5 | stumpy-37-12h-6 | stumpy-39-12h-1 | stumpy-39-12h-2 | stumpy-39-12h-3 | stumpy-39-12h-4 | stumpy-39-12h-5 | stumpy-39-12h-6 |
|---|---|---|---|---|---|---|---|---|---|---|---|---|---|---|---|
| 541_NEG | 8.133 | 259.02162 | D-Glucose-6-phosphate | 24592 | 19786 | 16899 | 19737 | 21032 | 20090 | 98926 | 95828 | 65570 | 76124 | 97947 | 81631 |
| 542_NEG | 7.96 | 259.02173 | D-Glucose-6-phosphate | 5997 | 2177 | 6202 | 3419 | 2855 | 2420 | 21064 | 15213 | 21083 | 26537 | 21413 | 19667 |
| 543_NEG | 3.017 | 259.03119 | D-Glucose-6-phosphate | 4360 | 2997 | 2614 | 2860 | 1888 | 2581 | 11476 | 9040 | 3840 | 4071 | 3333 | 4452 |
| 1151_POS | 7.706 | 261.03476 | D-Glucose-6-phosphate | 15439 | 8209 | 5806 | 5844 | 16846 | 5429 | 4647 | 9839 | 20431 | 7204 | 14265 | 4760 |
| 1152_POS | 7.927 | 261.03527 | D-Glucose-6-phosphate | 26539 | 37798 | 17571 | 15040 | 21541 | 25641 | 45860 | 14888 | 17597 | 30398 | 11937 | 16414 |
| | | | | 76927 | 70967 | 49092 | 46900 | 64162 | 56161 | 181973 | 144808 | 128521 | 144334 | 148895 | 126924 |
| 226_NEG | 7.272 | 171.00563 | Glycerol 3-phosphoate | 2958 | 941 | 1312 | 1763 | 2685 | 1280 | 12736 | 16331 | 6412 | 11443 | 6317 | 11016 |
| 227_NEG | 6.463 | 171.00671 | Glycerol 3-phosphoate | 153967 | 128374 | 135291 | 91084 | 126137 | 127253 | 415069 | 515794 | 382443 | 442951 | 508502 | 542567 |
| | | | | 156925 | 129315 | 136603 | 92847 | 128822 | 128533 | 427805 | 532125 | 388855 | 454394 | 514819 | 553583 |
| 570_NEG | 0.594 | 265.01056 | 1,3-Diphosphoglycerate | 14129 | 24605 | 27840 | 16415 | 16008 | 17724 | 11558 | 11508 | 13446 | 16112 | 12428 | 16513 |
| 263_NEG | 8.295 | 184.98568 | 3-Phosphoglycerate | 1211 | 2527 | 15546 | 5243 | 15858 | 19081 | 111642 | 56158 | 4614 | 5288 | 54965 | 2734 |
| 119_NEG | 1.445 | 133.002 | Malate | 27901 | 39192 | 21185 | 34426 | 17595 | 17840 | 33485 | 42157 | 43955 | 38205 | 76069 | 25494 |
| 1404_NEG | 4.993 | 419.21146 | Malate | 9770 | 3494 | 14796 | 3880 | 7428 | 10463 | 18990 | 13021 | 16014 | 10580 | 20738 | 18364 |
| 1405_NEG | 5.173 | 419.21258 | Malate | 5213 | 3518 | 10735 | 4879 | 5365 | 8242 | 22462 | 19248 | 24887 | 21625 | 25227 | 24899 |
| | | | | 42884 | 46204 | 46716 | 43185 | 30388 | 36545 | 74937 | 74426 | 84856 | 70410 | 122034 | 68757 |
| 1423_POS | 3.76 | 287.11117 | Fumarate | 16770 | 1668 | 2731 | 3702 | 349 | 2154 | 4854 | 4193 | 3704 | 6482 | 1884 | 2202 |
| 1424_POS | 4.996 | 287.11587 | Fumarate | 28467 | 10802 | 2741 | 5400 | 8728 | 10974 | 4148 | 15155 | 19889 | 7874 | 6812 | 7521 |
| | | | | 45237 | 12470 | 5472 | 9102 | 9077 | 13128 | 9002 | 19348 | 23593 | 14356 | 8696 | 9723 |
| 79_NEG | 6.509 | 117.01928 | Succinate | 4578 | 3198 | 3648 | 3098 | 3614 | 3956 | 23102 | 22241 | 17064 | 23279 | 22862 | 23648 |
| 6673_POS | 3.008 | 810.5941 | Acetyl CoA | 2126 | 474424 | 22959 | 172671 | 1358 | 1637 | 2993 | 2139 | 7896 | 7439 | 452 | 163405 |
| 6674_POS | 4.073 | 810.59534 | Acetyl CoA | 186 | 114251 | 4157 | 16672 | 429 | 783 | 1452 | 1099 | 1539 | 7623 | 209 | 10229 |
| 6675_POS | 1.224 | 810.59568 | Acetyl CoA | 22886 | 802355 | 122810 | 802717 | 7489 | 16153 | 32395 | 13093 | 24561 | 67266 | 7707 | 288033 |
| 6676_POS | 3.413 | 810.59593 | Acetyl CoA | 1320 | 1093792 | 22940 | 167446 | 1026 | 4526 | 5398 | 1424 | 3070 | 25900 | 432 | 108413 |
| 6677_POS | 1.955 | 810.5966 | Acetyl CoA | 44617 | 218966 | 440092 | 289323 | 11856 | 27401 | 47303 | 22854 | 401681 | 118184 | 41724 | 218711 |
| 6678_POS | 1.035 | 810.59843 | Acetyl CoA | 22129 | 3123193 | 249521 | 6555918 | 68164 | 172249 | 99642 | 155557 | 75341 | 275540 | 9170 | 595889 |
| 6679_POS | 0.823 | 810.60047 | Acetyl CoA | 1248719 | 2519208 | 430975 | 1331393 | 67066 | 87892 | 193678 | 67372 | 297422 | 3728773 | 107765 | 4561860 |
| | | | | 1341983 | 8346189 | 1293454 | 9336140 | 157388 | 310641 | 382861 | 263538 | 811510 | 4230725 | 167459 | 5946540 |
| 81_NEG | 6.471 | 118.05102 | Threonine | 6332 | 8335 | 5326 | 3310 | 9083 | 4984 | 40744 | 25697 | 13863 | 23121 | 28646 | 23638 |
| 66_POS | 4.22 | 102.0474 | Threonine | 8813 | 5928 | 5667 | 4894 | 5338 | 6146 | 5230 | 6140 | 8052 | 4983 | 2760 | 5254 |
| 142_POS | 6.62 | 120.10086 | Threonine | 46051 | 163817 | 271040 | 230913 | 99993 | 65608 | 537450 | 68270 | 55940 | 174742 | 134720 | 238224 |

| | | | | | | | | | | | | | | | |
|---|---|---|---|---|---|---|---|---|---|---|---|---|---|---|---|
| 143_POS | 6.21 | 120.10095 | Threonine | 297426 | 173505 | 253967 | 206630 | 83875 | 83213 | 466376 | 59746 | 60791 | 587689 | 99520 | 181699 |
| 144_POS | 5.947 | 120.10102 | Threonine | 244627 | 1563975 | 1016700 | 635168 | 855168 | 879402 | 811640 | 830386 | 821323 | 297440 | 190495 | 1868137 |
| 145_POS | 5.723 | 120.10132 | Threonine | 928001 | 1805505 | 3094337 | 2297925 | 695355 | 688744 | 5836474 | 570516 | 537165 | 2869470 | 864786 | 2322284 |
| 146_POS | 5.616 | 120.10166 | Threonine | 1072899 | 1689072 | 2340949 | 1857165 | 693931 | 651772 | 3875907 | 391077 | 488855 | 2225356 | 785021 | 1772579 |
| | | | | 2604149 | 5410137 | 6987986 | 5236005 | 2442743 | 2379869 | 11573821 | 1951832 | 1985989 | 6182801 | 2105948 | 6411815 |
| 280_NEG | 7.247 | 191.0194 | Citrate | 21530 | 18384 | 15904 | 8900 | 10958 | 15105 | 8577 | 20261 | 36713 | 39316 | 16317 | 31527 |
| 283_NEG | 8.249 | 191.02056 | Citrate | 2809372 | 1060141 | 2552015 | 984434 | 886810 | 1818024 | 1510532 | 1403952 | 3064857 | 3265746 | 1576496 | 2353796 |
| 285_NEG | 8.351 | 191.02098 | Citrate | 416717 | 1067655 | 2552015 | 984434 | 224266 | 1818024 | 196839 | 1399245 | 3064857 | 648141 | 625125 | 662147 |
| 1549_NEG | 0.59 | 459.19968 | Citrate | 831 | 2072 | 2920 | 727 | 719 | 687 | 3421 | 1175 | 2095 | 1952 | 1179 | 5116 |
| 1550_NEG | 7.088 | 459.20629 | Citrate | 3195 | 3490 | 9350 | 5476 | 7803 | 7765 | 10290 | 10558 | 20486 | 7751 | 9165 | 10105 |
| 64_NEG | 9.629 | 111.00895 | Citrate | 29590 | 20897 | 60881 | 9473 | 11906 | 8396 | 10065 | 17085 | 15448 | 31537 | 12783 | 79574 |
| 65_NEG | 8.788 | 111.00915 | Citrate | 126351 | 85401 | 70120 | 24685 | 73259 | 47176 | 71601 | 48549 | 82467 | 59150 | 64605 | 53945 |
| 589_POS | 1.218 | 191.10148 | Citrate | 11495 | 0 | 973 | 0 | 7446 | 1973 | 1859 | 15050 | 3653 | 243 | 2871 | 0 |
| 3144_POS | 4.2 | 465.16799 | Citrate | 159621 | 137788 | 193244 | 188307 | 217771 | 130076 | 188369 | 208418 | 167683 | 198874 | 228496 | 154565 |
| | | | | 3578702 | 2395828 | 5457422 | 2206436 | 1440938 | 3847226 | 2001553 | 3124293 | 6458259 | 4252710 | 2537037 | 3350775 |
| 281_NEG | 9.634 | 191.02004 | Isocitrate | 435486 | 124109 | 71613 | 9869 | 34112 | 36375 | 18029 | 103566 | 228735 | 304630 | 92034 | 248141 |
| 282_NEG | 8.896 | 191.0202 | Isocitrate | 353168 | 224641 | 160046 | 50067 | 335925 | 226866 | 190584 | 67635 | 181139 | 242698 | 105884 | 222013 |
| 284_NEG | 8.781 | 191.02083 | Isocitrate | 647857 | 297114 | 377808 | 146403 | 532732 | 175087 | 226816 | 143916 | 469038 | 361957 | 227852 | 169011 |
| | | | | 1436511 | 645864 | 609467 | 206339 | 902769 | 438328 | 435429 | 315117 | 878912 | 909285 | 425770 | 639165 |
| 697_NEG | 0.543 | 288.10608 | alpha-Ketoglutarate | 6838 | 3065 | 4111 | 3390 | 3252 | 5275 | 3406 | 4101 | 6148 | 4052 | 3992 | 3974 |
| 1036_NEG | 6.427 | 346.15973 | alpha-Ketoglutarate | 3763 | 6186 | 15076 | 7545 | 12425 | 9699 | 20600 | 20817 | 37547 | 20902 | 21517 | 24009 |
| 1037_NEG | 6.14 | 346.16033 | alpha-Ketoglutarate | 4351 | 5152 | 12899 | 7648 | 10844 | 6248 | 14385 | 14344 | 22047 | 12843 | 12868 | 14857 |
| 1038_NEG | 6.269 | 346.16056 | alpha-Ketoglutarate | 4027 | 3793 | 33246 | 10309 | 24734 | 15360 | 18049 | 16635 | 36447 | 14247 | 17284 | 17946 |
| 362_POS | 4.521 | 156.04099 | alpha-Ketoglutarate | 139083 | 98963 | 100017 | 88303 | 61726 | 58613 | 45632 | 58922 | 79949 | 89785 | 67298 | 73519 |
| 363_POS | 4.312 | 156.04629 | alpha-Ketoglutarate | 25470 | 4556 | 3120 | 6607 | 625 | 797 | 6012 | 7506 | 13444 | 9740 | 6996 | 6789 |
| 1984_POS | 6.813 | 346.15771 | alpha-Ketoglutarate | 12363 | 7227 | 4592 | 3377 | 7773 | 6517 | 8225 | 9635 | 8662 | 8296 | 5734 | 8760 |
| | | | | 195895 | 128942 | 173061 | 127179 | 121379 | 102509 | 116309 | 131960 | 204244 | 159865 | 135689 | 149854 |
| 42_NEG | 5.922 | 88.04027 | Alanine | 6560 | 4110 | 4323 | 8480 | 10399 | 7422 | 51180 | 35309 | 57030 | 43573 | 52667 | 33408 |
| 39_POS | 6.219 | 88.03754 | Alanine | 11267 | 10147 | 10304 | 10371 | 7745 | 10600 | 9491 | 7236 | 7793 | 11094 | 7782 | 8148 |
| 40_POS | 6.51 | 88.03879 | Alanine | 34023 | 22093 | 23664 | 24342 | 14719 | 24203 | 17051 | 17350 | 19117 | 26072 | 14202 | 17819 |
| | | | | 51850 | 36350 | 38291 | 43193 | 32863 | 42225 | 77722 | 59895 | 83940 | 80739 | 74651 | 59375 |
| 150_NEG | 6.696 | 146.04599 | Glutamate | 61449 | 47258 | 96566 | 74711 | 87523 | 50153 | 375221 | 465361 | 291067 | 389932 | 429096 | 438284 |
| 320_POS | 7.35 | 148.05917 | Glutamate | 11358 | 7127 | 8276 | 9839 | 7532 | 7081 | 10221 | 9089 | 8230 | 8963 | 15827 | 10769 |

| | | | | | | | | | | | | | | | |
|---|---|---|---|---|---|---|---|---|---|---|---|---|---|---|---|
| 321_POS | 6.405 | 148.06024 | Glutamate | 260784 | 216145 | 219414 | 244208 | 126823 | 217101 | 138399 | 113157 | 145773 | 262155 | 124071 | 122061 |
| 187_POS | 7.996 | 130.04842 | Glutamate | 8566 | 4852 | 9219 | 5671 | 11756 | 8098 | 9229 | 11281 | 9889 | 6738 | 10038 | 11834 |
| | | | | 342157 | 275382 | 333475 | 334429 | 233634 | 282433 | 533070 | 598888 | 454959 | 667788 | 579032 | 582948 |
| 70_NEG | 5.287 | 114.05592 | Proline | 18526 | 9083 | 10711 | 13093 | 13080 | 16851 | 115765 | 103700 | 83262 | 74348 | 120137 | 125607 |
| 402_NEG | 3.979 | 231.13444 | Proline | 11019 | 4126 | 46207 | 5842 | 16535 | 13303 | 24226 | 20769 | 25352 | 23404 | 21075 | 24605 |
| 110_POS | 0.901 | 114.05374 | Proline | 6909 | 282 | 200 | 1750 | 10240 | 11218 | 2697 | 13115 | 13518 | 575 | 2735 | 0 |
| 119_POS | 1.214 | 116.06883 | Proline | 26003 | 0 | 645 | 742 | 5907 | 6462 | 5382 | 12459 | 7723 | 3410 | 3681 | 334 |
| 120_POS | 8.754 | 116.06921 | Proline | 99298 | 55732 | 69022 | 62541 | 47359 | 67711 | 28995 | 48552 | 67540 | 80919 | 51161 | 23176 |
| 121_POS | 1.889 | 116.0695 | Proline | 6897 | 0 | 971 | 0 | 38515 | 5569 | 3161 | 17351 | 7638 | 3267 | 8258 | 470 |
| 122_POS | 0.966 | 116.0699 | Proline | 5237 | 0 | 303 | 0 | 13562 | 2293 | 3861 | 21206 | 2935 | 448 | 2743 | 0 |
| 123_POS | 5.127 | 116.07059 | Proline | 3846067 | 3180868 | 3652061 | 3127209 | 2708176 | 3463998 | 2882216 | 2755357 | 2870119 | 3729522 | 3076691 | 2757982 |
| 875_POS | 1.918 | 231.13239 | Proline | 29596 | 1870 | 3649 | 154 | 14867 | 7012 | 5994 | 20038 | 16471 | 11376 | 7968 | 2303 |
| 876_POS | 5.135 | 231.13282 | Proline | 17634 | 10827 | 8519 | 12826 | 7295 | 10853 | 7420 | 8827 | 9342 | 11727 | 7510 | 6703 |
| | | | | 4067186 | 3262788 | 3792288 | 3224157 | 2875536 | 3605270 | 3079717 | 3021374 | 3103900 | 3938996 | 3301959 | 2941180 |
| 12_POS | 6.509 | 74.02297 | Glycine | 12998 | 8757 | 9952 | 9428 | 6847 | 9885 | 8018 | 7533 | 7756 | 9356 | 6080 | 8331 |

**Supplementary Table 3** Primers used in PAD1-P2A-tdTomato plamid construction.

| Primer | Sequence |
|---|---|
| PAD1-3'UTR-1F | aactcaactgcaacgaagcttAGCTTAGGGGAGCCAGTGGA |
| PAD1-3'UTR-1R | tgaaagCTCGAGAACGAGAGCTAAGTGAAG |
| PAD1-CDS-1F | ctctcgttctcgagCTTTCAAATGGTTTCACAGCGG |
| PAD1-CDS-1R | gcccttgctcaccatactagtTTGCGGAGCAGCCTCACG |

**Supplementary Table 4** Primers used in gene expression via RT-qPCR.

| Gene | TriTrypDB ID | Forward primer (5'-3‘) | Reverse primer (5'-3‘) |
|---|---|---|---|
| *28s rRNA* | Tb927.7.6885<br>Tb927.6.184<br>Tb927.3.3452<br>Tb927.3.3443<br>Tb927.3.3434<br>Tb927.3.3425<br>Tb927.2.1975<br>Tb927.2.1510<br>Tb927.2.1416 | AGGGATAACTGGCTTGTGGC | ACCTGTCTCACGACGGTCTA |
| *Endonuclease G* | Tb927.8.4040<br>Tb927.8.4090 | AGGACCACTGCTGCTGATTC | CTCTTGTGAAACTGTGCGGC |
| *Metacasp-3* | Tb927.6.930 | GTTGCCACTCAGGCTCCAT | GCCTGTAGAACCCGTACCAAA |
| *tSNAP42* | Tb927.5.3910 | CGGGGAAAAACGACAAAACGA | GCTTCACCCACACATACGGA |
| *TbAtg8.1* | Tb927.7.5900 | GCGATTGAATGAATCCGCAAAG | CGTAGCACGCTGACAAACTG |
| *TbAtg8.2* | Tb927.7.5910 | CACCTTTGAGAGCCGACAGT | AGAGTGCAGATTCGGCTTCC |

**Supplementary Table 5** Pairwise multiple comparisons (*p*-values) from trypanosomes thermosensitivity experiments.

Raw *p*-values

| 24hr | LS 27°C | LS 37°C | LS 38°C | LS 39°C | SS 27°C | SS 37°C | SS 38°C |
|---|---|---|---|---|---|---|---|
| LS 37°C | 0.006 | | | | | | |
| LS 38°C | 0.0417 | 0.0066 | | | | | |
| LS 39°C | 0.0002 | 0.0046 | <0.0001 | | | | |
| SS 27°C | 0.0046 | 0.0069 | 0.0177 | 0.0003 | | | |
| SS 37°C | 0.5455 | 0.005 | 0.3363 | 0.0042 | 0.0164 | | |
| SS 38°C | 0.0087 | 0.0052 | 0.0018 | 0.0028 | 0.0007 | 0.0263 | |
| SS 39°C | 0.0036 | 0.005 | 0.0019 | 0.3317 | 0.0028 | 0.0118 | 0.0156 |

| 48hr | LS 27°C | LS 37°C | LS 38°C | LS 39°C | SS 27°C | SS 37°C | SS 38°C |
|---|---|---|---|---|---|---|---|
| LS 37°C | 0.0001 | | | | | | |
| LS 38°C | 0.267 | <0.0001 | | | | | |
| LS 39°C | 0.0059 | 0.0001 | 0.0101 | | | | |
| SS 27°C | 0.0011 | 0.0001 | 0.0123 | 0.0014 | | | |
| SS 37°C | 0.0337 | 0.0001 | 0.041 | 0.0024 | 0.0008 | | |
| SS 38°C | 0.0056 | 0.0001 | 0.0123 | 0.0128 | 0.0007 | 0.0006 | |
| SS 39°C | 0.0059 | 0.0001 | 0.0101 | >0.9999 | 0.0014 | 0.0024 | 0.0128 |

| 72hr | LS 27°C | LS 37°C | LS 38°C | LS 39°C | SS 27°C | SS 37°C | SS 38°C |
|---|---|---|---|---|---|---|---|
| LS 37°C | 0.0049 | | | | | | |
| LS 38°C | 0.1378 | 0.0049 | | | | | |
| LS 39°C | 0.0063 | 0.0049 | 0.0291 | | | | |
| SS 27°C | 0.0009 | 0.005 | 0.0006 | 0.0022 | | | |
| SS 37°C | 0.01 | 0.0049 | 0.0816 | 0.0036 | 0.0023 | | |
| SS 38°C | 0.006 | 0.0049 | 0.0318 | 0.0634 | 0.0021 | 0.0007 | |
| SS 39°C | 0.0063 | 0.0049 | 0.0291 | >0.9999 | 0.0022 | 0.0036 | 0.0634 |

| 96hr | LS 27°C | LS 37°C | LS 38°C | LS 39°C | SS 27°C | SS 37°C | SS 38°C |
|---|---|---|---|---|---|---|---|
| LS 37°C | 0.0014 | | | | | | |
| LS 38°C | 0.0225 | 0.0014 | | | | | |
| LS 39°C | 0.0192 | 0.0014 | 0.039 | | | | |
| SS 27°C | 0.0023 | 0.0014 | 0.0026 | 0.0038 | | | |
| SS 37°C | 0.0192 | 0.0014 | 0.039 | >0.9999 | 0.0038 | | |
| SS 38°C | 0.0192 | 0.0014 | 0.039 | >0.9999 | 0.0038 | >0.9999 | |
| SS 39°C | 0.0192 | 0.0014 | 0.039 | >0.9999 | 0.0038 | >0.9999 | >0.9999 |

FDR-adjusted *p*-values

| 24hr | LS 27°C | LS 37°C | LS 38°C | LS 39°C | SS 27°C | SS 37°C | SS 38°C |
|---|---|---|---|---|---|---|---|
| LS 37°C | 0.0106 | | | | | | |
| LS 38°C | 0.0467 | 0.0108 | | | | | |
| LS 39°C | 0.0029 | 0.0096 | 0.0019 | | | | |
| SS 27°C | 0.0096 | 0.0108 | 0.0216 | 0.0029 | | | |
| SS 37°C | 0.5455 | 0.0096 | 0.3488 | 0.0096 | 0.0209 | | |
| SS 38°C | 0.0129 | 0.0096 | 0.0088 | 0.0096 | 0.0052 | 0.0307 | |
| SS 39°C | 0.0096 | 0.0096 | 0.0088 | 0.3488 | 0.0096 | 0.0165 | 0.0208 |

| 48hr | LS 27°C | LS 37°C | LS 38°C | LS 39°C | SS 27°C | SS 37°C | SS 38°C |
|---|---|---|---|---|---|---|---|
| LS 37°C | 0.0005 | | | | | | |
| LS 38°C | 0.2769 | 0.0005 | | | | | |
| LS 39°C | 0.0091 | 0.0005 | 0.0142 | | | | |
| SS 27°C | 0.0027 | 0.0005 | 0.0149 | 0.0031 | | | |
| SS 37°C | 0.0378 | 0.0005 | 0.0441 | 0.0046 | 0.0023 | | |
| SS 38°C | 0.0091 | 0.0005 | 0.0149 | 0.0149 | 0.0022 | 0.0021 | |
| SS 39°C | 0.0091 | 0.0005 | 0.0142 | >0.9999 | 0.0031 | 0.0046 | 0.0149 |

| 72hr | LS 27°C | LS 37°C | LS 38°C | LS 39°C | SS 27°C | SS 37°C | SS 38°C |
|---|---|---|---|---|---|---|---|
| LS 37°C | 0.0087 | | | | | | |
| LS 38°C | 0.1429 | 0.0087 | | | | | |
| LS 39°C | 0.0092 | 0.0087 | 0.037 | | | | |
| SS 27°C | 0.0083 | 0.0087 | 0.0083 | 0.0087 | | | |
| SS 37°C | 0.014 | 0.0087 | 0.0879 | 0.0087 | 0.0087 | | |
| SS 38°C | 0.0092 | 0.0087 | 0.0387 | 0.071 | 0.0087 | 0.0083 | |
| SS 39°C | 0.0092 | 0.0087 | 0.037 | >0.9999 | 0.0087 | 0.0087 | 0.071 |

| 96hr | LS 27°C | LS 37°C | LS 38°C | LS 39°C | SS 27°C | SS 37°C | SS 38°C |
|---|---|---|---|---|---|---|---|
| LS 37°C | 0.0056 | | | | | | |
| LS 38°C | 0.0351 | 0.0056 | | | | | |
| LS 39°C | 0.0316 | 0.0056 | 0.0497 | | | | |
| SS 27°C | 0.008 | 0.0056 | 0.008 | 0.0081 | | | |
| SS 37°C | 0.0316 | 0.0056 | 0.0497 | >0.9999 | 0.0081 | | |
| SS 38°C | 0.0316 | 0.0056 | 0.0497 | >0.9999 | 0.0081 | >0.9999 | |
| SS 39°C | 0.0316 | 0.0056 | 0.0497 | >0.9999 | 0.0081 | >0.9999 | >0.9999 |
